# Multi-omics insights into the biological mechanisms underlying statistical gene-by-lifestyle interactions with smoking and alcohol consumption

**DOI:** 10.3389/fgene.2022.954713

**Published:** 2022-12-05

**Authors:** Timothy D. Majarian, Amy R. Bentley, Vincent Laville, Michael R. Brown, Daniel I. Chasman, Paul S. de Vries, Mary F. Feitosa, Nora Franceschini, W. James Gauderman, Casey Marchek, Daniel Levy, Alanna C. Morrison, Michael Province, Dabeeru C. Rao, Karen Schwander, Yun Ju Sung, Charles N. Rotimi, Hugues Aschard, C. Charles Gu, Alisa K. Manning, on behalf of the CHARGE Gene-Lifestyle Interactions Working Group

**Affiliations:** ^1^ Program in Metabolism, Broad Institute of MIT and Harvard, Cambridge, MA, United States; ^2^ Center for Research on Genomics and Global Health, National Human Genome Research Institute, US National Institutes of Health, Bethesda, MD, United States; ^3^ Department of Computational Biology, Institut Pasteur, Université Paris Cité, Paris, France; ^4^ Human Genetics Center, Department of Epidemiology, Human Genetics, and Environmental Sciences, School of Public Health, The University of Texas Health Science Center at Houston, Houston, TX, United States; ^5^ Division of Preventive Medicine, Brigham and Women’s Hospital, Harvard Medical School, Boston, MA, United States; ^6^ Division of Statistical Genomics, Department of Genetics, Washington University School of Medicine, St. Louis, MO, United States; ^7^ Department of Epidemiology, Gillings School of Global Public Health, University of North Carolina at Chapel Hill, Chapel Hill, NC, United States; ^8^ Biostatistics, Department of Preventive Medicine, University of Southern California, Los Angeles, CA, United States; ^9^ Clinical and Translational Epidemiology Unit, Massachusetts General Hospital, Boston, MA, United States; ^10^ The Population Sciences Branch, National Heart, Lung, and Blood Institute, National Institutes of Health, Bethesda, MA, United States; ^11^ Division of Biostatistics, Washington University School of Medicine, St. Louis, MO, United States; ^12^ Program in Genetic Epidemiology and Statistical Genetics, Harvard T.H. Chan School of Public Health, Boston, MA, United States; ^13^ Department of Medicine and Harvard Medical School, Boston, MA, United States

**Keywords:** multi-omics, gene-lifestyle interactions, smoking, alcohol, serum lipids, blood pressure, summary data

## Abstract

Though both genetic and lifestyle factors are known to influence cardiometabolic outcomes, less attention has been given to whether lifestyle exposures can alter the association between a genetic variant and these outcomes. The Cohorts for Heart and Aging Research in Genomic Epidemiology (CHARGE) Consortium’s Gene-Lifestyle Interactions Working Group has recently published investigations of genome-wide gene-environment interactions in large multi-ancestry meta-analyses with a focus on cigarette smoking and alcohol consumption as lifestyle factors and blood pressure and serum lipids as outcomes. Further description of the biological mechanisms underlying these statistical interactions would represent a significant advance in our understanding of gene-environment interactions, yet accessing and harmonizing individual-level genetic and ‘omics data is challenging. Here, we demonstrate the coordinated use of summary-level data for gene-lifestyle interaction associations on up to 600,000 individuals, differential methylation data, and gene expression data for the characterization and prioritization of loci for future follow-up analyses. Using this approach, we identify 48 genes for which there are multiple sources of functional support for the identified gene-lifestyle interaction. We also identified five genes for which differential expression was observed by the same lifestyle factor for which a gene-lifestyle interaction was found. For instance, in gene-lifestyle interaction analysis, the T allele of rs6490056 (*ALDH2*) was associated with higher systolic blood pressure, and a larger effect was observed in smokers compared to non-smokers. In gene expression studies, this allele is associated with decreased expression of *ALDH2*, which is part of a major oxidative pathway. Other results show increased expression of *ALDH2* among smokers. Oxidative stress is known to contribute to worsening blood pressure. Together these data support the hypothesis that rs6490056 reduces expression of *ALDH2*, which raises oxidative stress, leading to an increase in blood pressure, with a stronger effect among smokers, in whom the burden of oxidative stress is greater. Other genes for which the aggregation of data types suggest a potential mechanism include: *GCNT4*×current smoking (HDL), *PTPRZ1*×ever-smoking (HDL), *SYN2*×current smoking (pulse pressure), and *TMEM116*×ever-smoking (mean arterial pressure). This work demonstrates the utility of careful curation of summary-level data from a variety of sources to prioritize gene-lifestyle interaction loci for follow-up analyses.

## 1 Introduction

Lifestyle and environmental exposures have been shown to modify the associations of common genetic variants with traits linked to cardiometabolic disease (Grarup et al., 2008; Montasser et al., 2009; Higashibata et al., 2012; Manning et al., 2012; Sung et al., 2014). Recent large-scale studies have expanded the genetic architecture of interaction effects with genome-wide interaction analyses with tens of thousands of people from diverse ancestral backgrounds (Feitosa et al., 2018; Sung et al., 2018; Bentley et al., 2019; de Vries et al., 2019; Sung et al., 2019). These studies implicate genetic loci for which the genetic effect on a phenotype of interest is modified by either alcohol consumption or cigarette smoking. These lifestyle exposures could impact the gene regulatory mechanisms through which the trait-associated alleles influence gene expression. Statistical tests that evaluate these models would require human cohorts with exposure and outcome data, genetic data, and methylation, gene expression or other ‘omics data. The process of finding, obtaining, harmonizing, and analyzing individual-level data from human cohorts presents a challenge, as these data are ‘controlled-access’ and require numerous regulatory steps for the researcher, the researcher’s institution and the entity managing access to the data. On the other hand, it is now common to publish summary-level association statistics from human cohorts. Therefore, an analysis of both genetic and non-genetic data that relies on summary data alone is particularly valuable in this context.

To this end, we leveraged multiple summary-level association datasets, both genetic and epigenomic, to evaluate possible mechanisms of gene-environment interactions effects. We use data from the CHARGE Consortium’s Gene-Lifestyle Interactions Working Group (Rao et al., 2017), published in five recent papers (Feitosa et al., 2018; Sung et al., 2018; Bentley et al., 2019; de Vries et al., 2019; Sung et al., 2019) together with differential methylation and gene expression data (Lonsdale et al., 2013; Dekkers et al., 2016; Huan et al., 2016; Joehanes et al., 2016; Parker et al., 2017; Richard et al., 2017; Liu et al., 2018; Nikodemova et al., 2018). We demonstrate that by considering diverse epigenetic associations at the same genetic loci and linking multiple forms of evidence to a common gene, gene-environment interaction effects may be more fully characterized and prioritized for future follow-up analyses.

## 2 Methods

### 2.1 Gene-lifestyle interaction (GLI) summary statistics

Summary statistics from four genome-wide interaction studies performed within the Cohorts for Heart and Aging Research in Genomic Epidemiology (CHARGE) consortium were gathered, covering blood pressure and lipid trait measures and cigarette smoking and alcohol consumption environmental exposures (Feitosa et al., 2018; Sung et al., 2018; de Vries et al., 2019; Sung et al., 2019). Each of these projects used the following GLI model: Y = β_0_ + β_G_ G + β_C_C + β_L_ L + β_GL_ G × L, where G represents the genetic variant, C the covariates (age, sex, and field center, where appropriate), and L the lifestyle exposure. The GLI models used additive allele effects and produced genome-wide joint tests of main effects and interaction effects. Sample sizes ranged from 175,000 to 602,000 individuals of multiple, self-identified ancestries (Supplemental Table S1).

We extracted individual variants with interaction *p*-value in any model less than 5 × 10^−5^ from each study for four subgroups, defined by the CHARGE Gene-Lifestyle Interaction Working Group: European ancestry (EA), African ancestry (AA), Hispanic ancestry (HA) and Asian Ancestry (ASA). Variants from trans-ancestry meta-analyses were also extracted for each project (Supplemental Table S1). Lipid trait measures included high-density lipoprotein cholesterol (HDL), low-density lipoprotein cholesterol (LDL), and triglycerides (TG). Four blood pressure traits were used: systolic blood pressure (SBP), diastolic BP (DBP), mean arterial pressure (MAP), and pulse pressure (PP). Environmental exposures were defined as: current drinking (yes/no), regular drinking (≥2 drinks per week/<2 drinks per week), drinking habits (≥8 glasses per week [heavy]/< 8 glasses per week [light]), current smoking (yes/no regular smoking in the past year), and ever smoked (yes/no 100 cigarettes smoked in lifetime).

Given the minor differences in filtering strategies for each of these projects, all meta-analysis results were re-processed using a common pipeline, as is described in more detail in (Laville et al., 2022). Briefly, SNPs were excluded for low MAF (<1%) or significant heterogeneity across included cohorts (*p* < 10–6). Meta-analyses were first conducted within ancestry groups, and these ancestry-specific results were meta-analyzed to produce the trans-ancestry meta-analysis results. All meta-analyses were implemented in METAL using the inverse variance scheme.

### 2.2 Epigenomic and transcriptomic data

Data describing the molecular signatures of the two environmental exposures were gathered from the literature, focusing on epigenomics and transcriptomics from population-based cohort studies (Table 1). For each of these sources, we used the statistical significance as defined within the publication or resource to determine which association results to use in our analysis. We used differential gene expression analysis (DExpr analysis) from studies of cigarette smoking (Huan et al., 2016; Parker et al., 2017; Nikodemova et al., 2018) and alcohol consumption (Liu et al., 2018). For genes, locus boundaries were defined by the start of the first exon and the end of the final exon, regardless of transcript, extended by 500 kb. Boundaries for DNA methylation (DNAm) sites were similarly defined by 500 kb on either side. Differential methylation analyses (DMe analysis) yield DNAm sites whose methylation is associated with a trait or exposure. These sites can be further associated with gene expression, defining expression quantitative trait methylation sites (eQTM). DNAm sites and eQTM were gathered from studies for lipids (Dekkers et al., 2016), blood pressure (Richard et al., 2017), cigarette smoking (Joehanes et al., 2016), and alcohol consumption (Liu et al., 2018), with some also providing mQTL analyses results (Richard et al., 2017; Liu et al., 2018). We also extracted significant expression quantitative trait loci (eQTL) from the Genetic Tissue-Expression (GTEx) project (Lonsdale et al., 2013) portal, version 8.

**TABLE 1:** Data used in locus prioritization.

Trait/exposure	Type	Source	Analysis	Sample size (ancestry^a^)	Data extracted
Blood Pressure/Smoking	Genetic Association	Sung *et al.* (2018), Sung *et al.* (2019)	GLI^b^	34,901 (AA); 162,370 (ASA); 380,612 (EA); 22,334 (HA)	464 SNPs
Lipids/Smoking	Genetic Association	Bentley *et al.* (2019)	GLI^b^	30,965 (AA); 116,570 (ASA); 237,477 (EA); 20,513 (HA)	218 SNPs
Blood Pressure/Alcohol	Genetic Association	Feitosa *et al.* (2018)	GLI^b^	26,458 (AA); 153,391 (ASA); 372,475 (EA); 17,433 (HA)	47 SNPs
Lipids/Alcohol	Genetic Association	de Vries *et al.* (2019)	GLI^b^	25,464 (AA); 120,881 (ASA); 226,505 (EA); 17,708 (HA)	168 SNPs
Blood Pressure & Lipids/Smoking & Alcohol	Genetic Association	Laville *et al.* (2022)	GLI^b^	Harmonized summary data of above GLI projects	Harmonized summary data of above GLI projects
Lipids	Methylation	Dekkers *et al.* (2016)	DMe	3,296 (EA)	28 DNAm
Blood pressure	Methylation	Richard *et al.* (2017)	DMe, eQTM	4,636 (AA); 11,345 (EA); 1,029 (HA)	126 DNAm, 13 eQTM
Smoking	Methylation	Joehanes *et al.* (2016)	DMe, eQTM	3,746 (AA); 12,161 (EA)	2,623 DNAm, 1,430 eQTM
Alcohol	Methylation	Liu *et al.* (2018)	DMe, eQTM	2,686 (AA); 10,231 (EA); 400 (HA)	328 DNAm, 14,160 eQTM
Smoking	Gene Expression	Parker *et al.* (2017)	DExpr	138 (AA); 377 (EA)	171 genes
Smoking	Gene Expression	Huan *et al.* (2016)	DExpr	10,233 (EA)	1,270 genes
Smoking	Gene Expression	Nikodemova *et al.* (2018)	DExpr	50 (NR)	25 genes
-	Gene Expression	Lonsdale *et al.* (2013)	eQTL	122 (AA); 12 (ASA); 802 (EA); 10 (NR)^3^	Genes associated with GLI SNPs from all tissues

^a^ Ancestry: African (AA), Asian (ASA), European (EA), Hispanic (HA), and Not Reported (NR).

^b^ Results from gene (G) × lifestyle (L) interaction model with covariates (C) age, sex, and field center (if appropriate): Y = β_0_ + β_G_ SNP + β_C_C + β_L_ L + β_GL_ SNP × L; 3Data drawn from GTEx, Portal (gtexportal.org accessed 7/21/2022).

### 2.3 Locus prioritization based on accumulation of evidence

**FIGURE 1:**
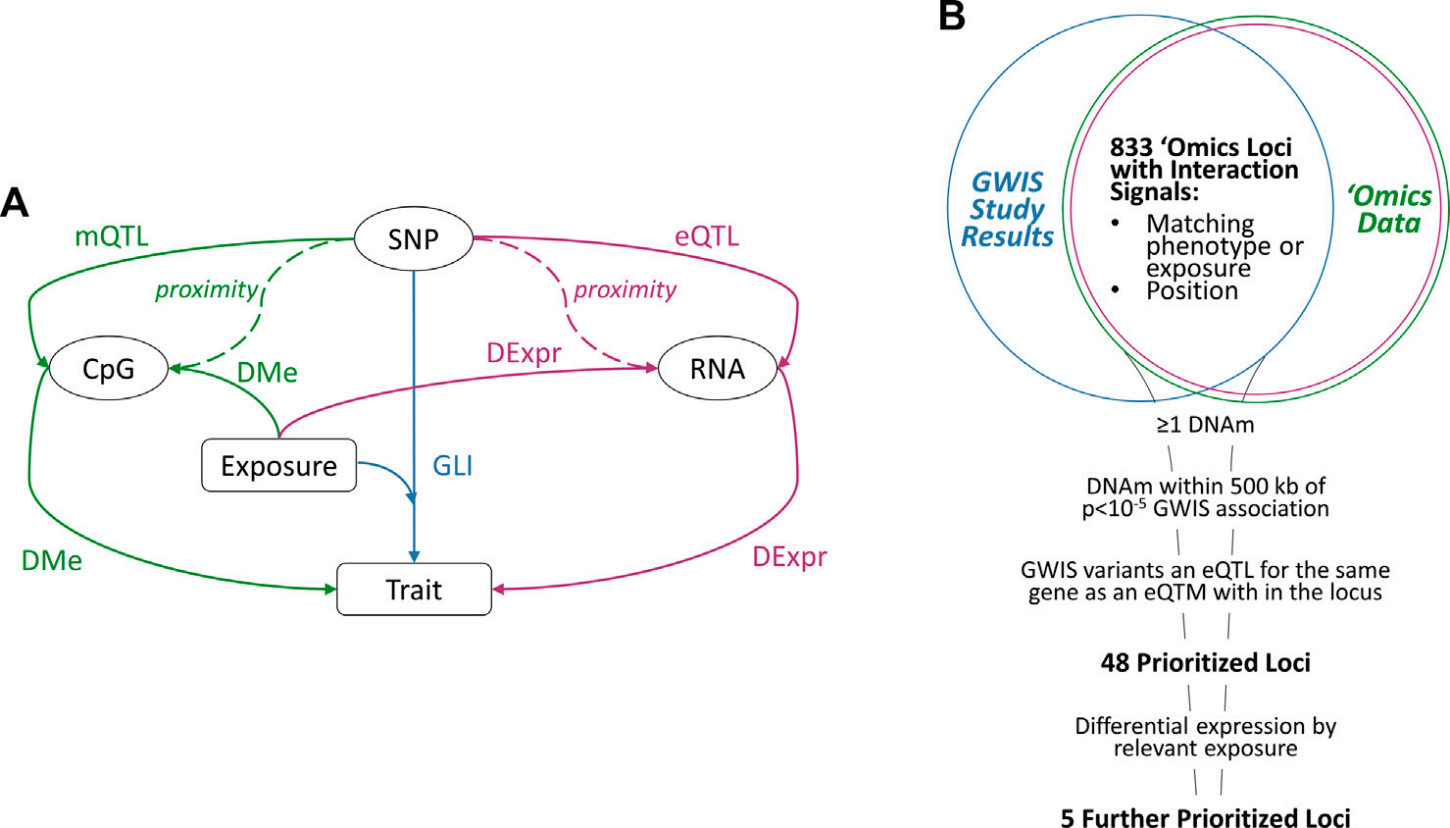
**(A)** Illustration of Possible Mechanisms Underlying Interaction Signals; rectangles represent study traits or measured lifestyle exposures; ovals represent three types of molecular risk factors: SNP genotype, RNA expression, and DNA methylation. Interactions are depicted by the “SNP-Exposure-Trait” path (GLI), with possible underlying molecular mechanisms shown along the sides for the transcriptomic (RNA expression) and epigenomic level (DNAm). The molecular effects are represented by solid arrows: expression QTL effect of a SNP allele on RNA expression levels (eQTL analysis) or methylation (mQTL analysis); differential methylation of a CpG site by trait or exposure (DMe analysis); and differential expression by trait or exposure (DExpr analysis). Dashed lines indicate physical proximity between elements. **(B)** Prioritization of GLI Loci; a panel of GLI loci from multiple genome-wide interaction studies (blue) were intersected with publicly-available epigenomic (green) and transcriptomic (purple) data for the relevant variants, lifestyle exposures, and traits. The resulting data were then prioritized using the listed criteria to yield 48 loci with multiple sources of molecular evidence underlying the interaction and five loci with differential expression by the exposure of interest.

Our prioritization schema consisted of several steps (Figure 1). First, we identified overlapping loci from the GLI association studies with DMe, mQTL and DExpr analyses. Starting from the set of GLI variants from the association studies (p < 5 × 10^−5^), we found significantly associated DNAm sites within 500 kb of the variants for the trait or exposure of interest. We next identified those genes for which both 1) the GLI variant was a significant eQTL and 2) the DNAm was a significant eQTM (“prioritized loci”). Finally, we selected genes from these prioritized loci that additionally had significant DExpr effects for the exposure of interest in the GLI association (“further prioritized loci”).

## 3 Results

### 3.1 Overview

We obtained genetic summary statistics from genome-wide interaction analyses of cigarette smoking habits and alcohol consumption with lipid traits (HDL, LDL, and TG) and blood pressure traits (SBP, DBP, MAP, and PP) (Table 1; Supplemental Table S1). The generation (Feitosa et al., 2018; Sung et al., 2018; Bentley et al., 2019; de Vries et al., 2019; Sung et al., 2019) and harmonization (Laville et al., 2020; Laville et al., 2022) of these summary statistics has been previously described, resulting in 140 sets of loci in four race/ancestry groups and one trans-ancestry meta-analysis (Supplementary Figure S1).

**FIGURE 2:**
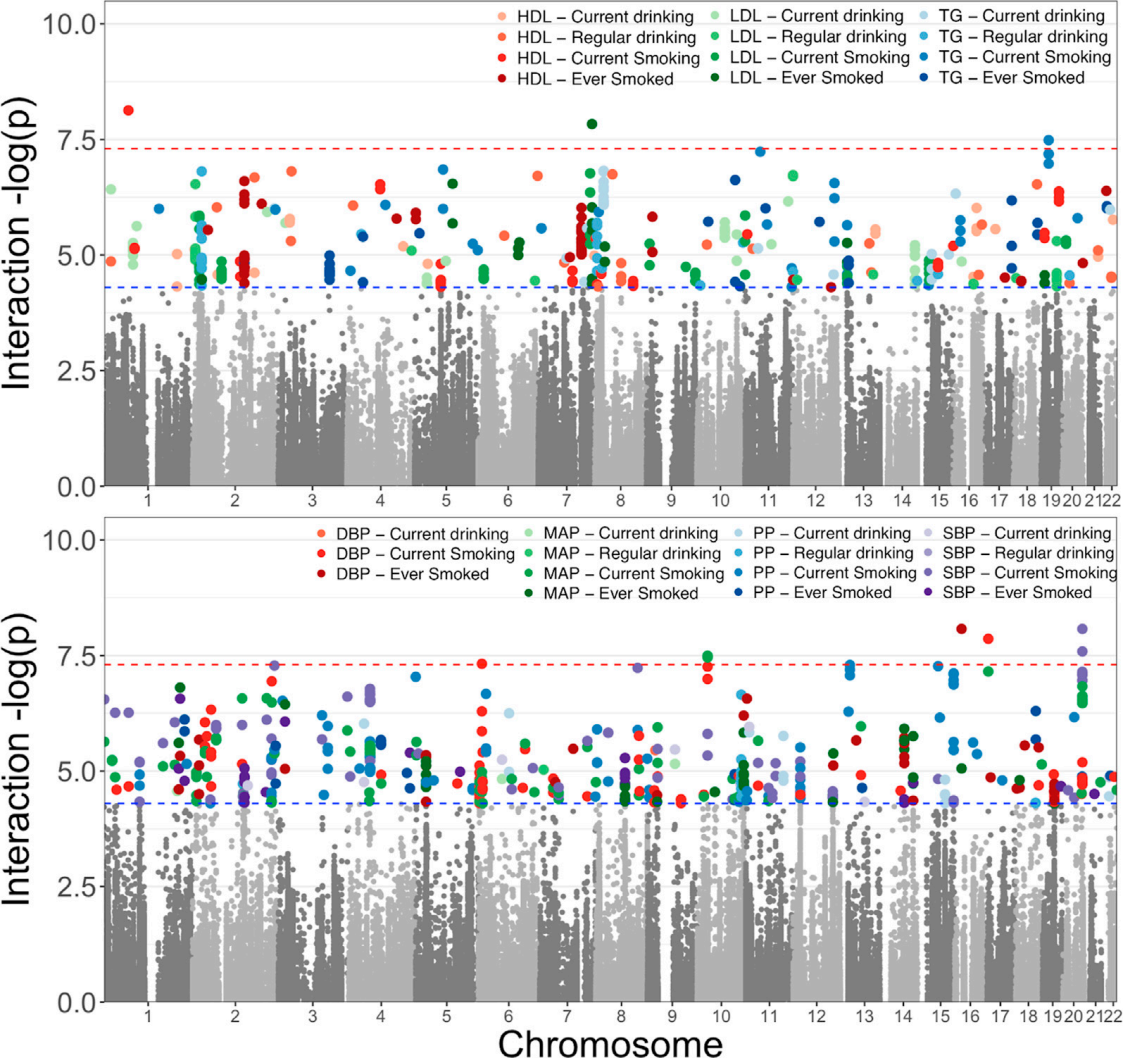
Selection of GLI Associations for Evaluation; a total of 897 variants were selected from meta-analyses of four ancestral groups and one trans-ancestry group of the variant interactions with two smoking and two alcohol exposure variables on three lipid measures (top figure) and four blood pressure traits (bottom figure). Here we show the most statistically significant associations across the five meta-analyses for each of the phenotype-exposure combinations. The results separated by ancestry group are available in Supplemental Figure 1.

**FIGURE 3:**
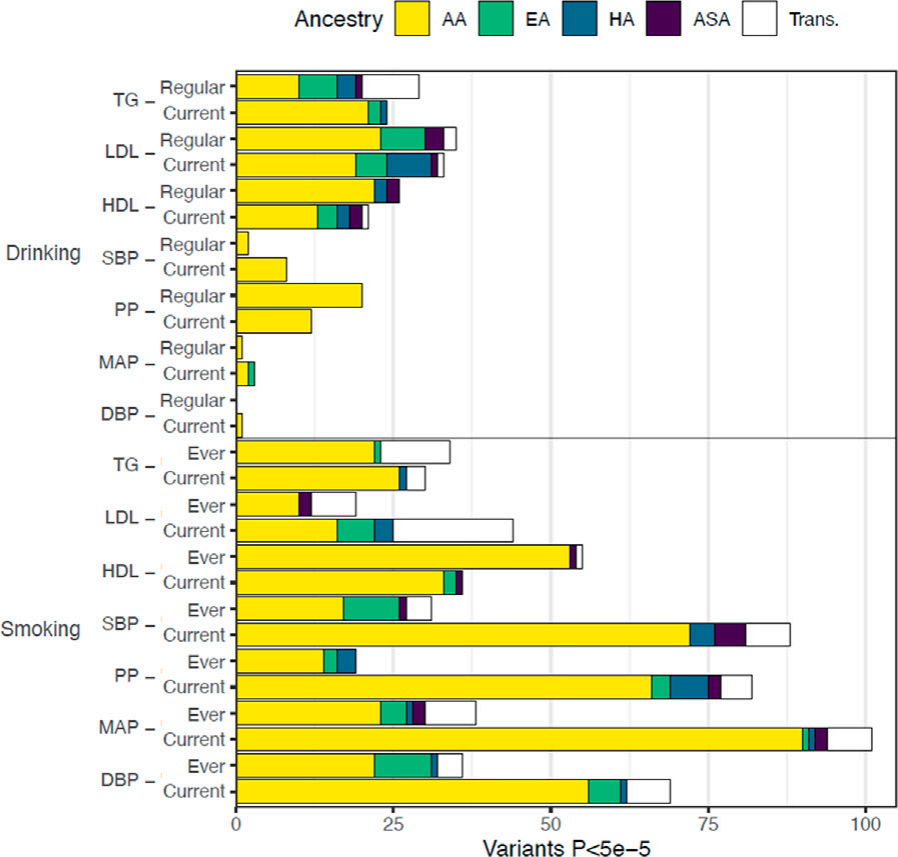
Distribution of GLI Associations across phenotypes and exposures. We display the total number of associated genetic variants discovered in each trait-exposure-ancestry group. The associations in models utilizing alcohol-related exposures are shown in the top figure and represent the minority of observed associations. The alcohol-related associates were dominated by analyses studying blood lipids. The associations related to cigarette smoking exposure are shown in the bottom figure with the majority of observations among analyses of blood pressure measures.

To restrict our efforts to the variants most likely to have both genetic associations and molecular associations, we considered genetic variants with an interaction *p*-value less than an arbitrary cut-off of 5 × 10^−5^ in the following model: Y = β_0_ + β_G_ G + β_C_C + β_L_ L + β_GL_ G × L, where G represents the genetic variant, C the covariates (age, sex, and field center, where appropriate), and L the lifestyle exposure. This filtering resulted in 897 variants (Figure 2). Of these variants, 682 were seen in smoking behavior interaction models, 511 were seen in models focusing on blood pressure traits, and 674 were observed in the analyses in the African ancestry subset (Figure 3).

### 3.2 Harmonization of association and omics data

We determined which of these variants also demonstrated significant epigenomic (methylation and gene-expression) associations with clinical outcomes, smoking behavior and/or alcohol consumption habits (Figure 1A). We used a variety of epigenetic association data from cohort studies, including differential methylation analysis (DMe analysis; see **Methods**) with both clinical outcomes (lipid traits, blood pressure traits) and lifestyle outcomes (smoking behaviors and alcohol consumption habits), and differential gene expression analysis (DExpr analysis; see **Methods**) with smoking behaviors only, as no expression data by alcohol consumption data were found at the time of our literature search (Table 1). For the traits and exposures considered in the genetic association analysis, we compiled a list of loci with significantly differentiated genes or differentially methylated methylation sites (DNAm) for the trait or exposure, extending the borders of the gene (defined by exon boundaries) or site by 500 KB. This intersection of genetic, epigenetic, and transcriptomic data resulted in 833 unique genes (Figure 1B).

#### 3.2.1 Prioritizing GLI associations

To further analyze these regions, we required loci to contain at least one DNAm site associated with expression of a gene (eQTM analysis; see **Methods**) and at least one of the GLI variants to be associated with the expression of the same gene in at least one tissue (eQTL analysis; see **Methods**). Using these criteria, we prioritized 48 loci. Of these, 37 loci were linked to smoking behavior and 28 loci were linked to lipid outcomes (Table 2). The majority of the prioritized loci were derived from analyses in individuals of African ancestry, a total of 30 loci, 26 of which were related to smoking behavior. These loci were evenly distributed between lipid and blood pressure traits. Our final prioritization step matched the lifestyle exposure from the GLI effect at the locus with significant genes from the DExpr analysis of the same exposure. At the 48 prioritized loci, five genes showed differential expression with a smoking exposure: *GCNT4*, *PTPRZ1*, *SYN2*, *ALDH2*, *TMEM116*. Each of these loci is discussed further below (Supplementary Figures S2–8) and details regarding the different types of evidence harmonized are available (Supplemental Tables S3–S9).

**TABLE 2:** Prioritized loci.

Exposure	Gene target	eQTL Tissue(s)	Number of GLI variants
*African Ancestry HDL*			
Curr Smk	*POLK*	Heart Left Ventricle, Pancreas, Thyroid^a^	5
Curr Smk	*GCNT4* ^b^	Artery Aorta, Testis	4
Curr Smk	*KLK8*	Skin Sun Exposed Lower leg	4
Curr Smk	*ANGPT1*	Thyroid	3
Curr Smk	*CARM1* ^c^	Muscle Skeletal	1
Curr Smk	*LIPC*	Liver, Pancreas	1
Curr Smk	*YIPF2* ^c^	Lung	1
Ev Smk	*PTPRZ1* ^b^ ^,^ ^c^	Artery Aorta	21
Ev Smk	*DNAH7*	Adipose Visceral Omentum, Pancreas, Thyroid^a^	1
*African Ancestry LDL*			
Ev Smk	*CRYGN*	Colon Sigmoid, Nerve Tibial	1
Ev Smk	*FRK*	Muscle Skeletal	1
Ev Smk	*WDR86*	Nerve Tibial	1
*African Ancestry TG*			
Curr Drnk	*BAHD1*	Colon Sigmoid, Stomach, Thyroid^a^	2
Curr Drnk	*DNAJC17*	Esophagus Mucosa	2
Ev Smk	*TRIO*	Ovary	1
*African Ancestry DBP*			
Ev Smk	*FNTB*	Muscle Skeletal, Skin Sun Exposed Lower leg	3
Ev Smk	*RAB15*	Adipose Subcutaneous, Nerve Tibial, Thyroid^a^	3
*African Ancestry MAP*			
Curr Smk	*CRTAC1*	Adipose Visceral Omentum, Artery Tibial	1
Curr Smk	*SULT4A1*	Colon Sigmoid	1
Ev Smk	*FNTB*	Muscle Skeletal, Skin Sun Exposed Lower leg	3
Ev Smk	*RAB15*	Adipose Subcutaneous, Nerve Tibial, Thyroid^a^	3
Ev Smk	*KCNK13*	Brain Hypothalamus	1
*African Ancestry PP*			
Curr Drnk	*MPI*	Artery Aorta, Lung, Whole Blood^a^	4
Curr Drnk	*SCAMP2*	Esophagus Mucosa, Pituitary, Whole Blood^a^	4
Curr Smk	*SYN2* ^b^	Artery Aorta	1
Ev Smk	*JMJD4*	Nerve Tibial	2
*African Ancestry SBP*			
Curr Smk	*CRTAC1*	Adipose Visceral Omentum, Artery Tibial	1
Ev Smk	*FNTB*	Muscle Skeletal, Skin Sun Exposed Lower leg	2
Ev Smk	*RAB15*	Adipose Subcutaneous, Nerve Tibial, Thyroid^a^	2
Ev Smk	*JMJD4*	Nerve Tibial	1
*Asian Ancestry HDL*			
Ev Smk	*KCTD10* ^c^	Artery Tibial, Skin Sun Exposed Lower leg	1
Ev Smk	*MY O 1H* ^c^	Lung	1
Ev Smk	*UBE3B* ^c^	Colon Transverse, Skin Sun Exposed Lower leg	1
*Asian Ancestry LDL*			
Drnk habits	*CPNE2*	Cells Transformed fibroblasts	3
Ev Smk	*KANK2* ^c^	Adipose Subcutaneous, Lung, Thyroid^a^	2
*Asian Ancestry TG*			
Drnk habits	*LIPC* ^c^	Thyroid	1
*Asian Ancestry MAP*			
Ev Smk	*ALDH2* ^b^ ^,^ ^c^	Esophagus Mucosa, Lung, Nerve Tibial, Thyroid^a^	2
Ev Smk	*TMEM116* ^b^ ^,^ ^c^	Artery Tibial, Heart Atrial Appendage, Whole Blood^a^	1
*Asian Ancestry SBP*			
Curr Smk	*ALDH2* ^b^ ^,^ ^c^	Esophagus Mucosa, Brain Cortex, Thyroid^a^	3
Ev Smk	*ALDH2* ^b^ ^,^ ^c^	Esophagus Mucosa, Skin Sun Exposed Lower leg	1
*European Ancestry LDL*			
Drnk habits	*APOC1* ^c^	Esophagus Mucosa	1
*European Ancestry TG*			
Drnk habits	*KRTCAP3* ^c^	Adrenal Gland, Thyroid, Whole Blood^a^	2
Drnk habits	*PPM1G* ^c^	Adipose Subcutaneous, Stomach, Thyroid^a^	2
*Hispanic Ancestry PP*			
Curr Smk	*STIM1*	Lung, Thyroid, Whole Blood	2
*Trans Ancestry LDL*			
Ev Smk	*CRYGN*	Colon Sigmoid, Nerve Tibial	1
Ev Smk	*WDR86*	Nerve Tibial	1
*Trans Ancestry TG*			
Drnk habits	*KRTCAP3* ^c^	Adrenal Gland, Thyroid, Whole Blood^a^	1
Drnk habits	*PPM1G* ^c^	Esophagus Muscularis, Muscle Skeletal	1

That the italic values indicate these are subheadings to identify the meta-analysis in which the results appeared. It could be described as the “Ancestry Trait Meta-Analysis” or just “Meta-Analysis”.

^a^ An eQTL, in multiple tissues: the most relevant 3 are shown (full results available in Supplemental Table S2).

^b^ Differential Expression by Exposure.

^c^ Loci with 2df GLI, association.

Abbreviations: Current Smoking (Curr Smk), Ever Smoking (Ev Smk), Drinking Habits (Drnk habits).

#### 3.2.2 Further prioritized loci

At the *GCNT4* locus, the common A allele at the rs3761743 variant was associated with decreased HDL levels among current smokers in an African-ancestry subset of cohorts, an effect that was attenuated among non-smokers (Interaction *p* = 1.5 × 10^−5^). *GCNT4* is a glycosyltransferase expressed primarily in the thymus (Schwientek et al., 2000) but rs3761743 was associated with decreased expression of GCNT4 in the aorta (*p* = 3.5 × 10^−6^) and increased expression in testis (*p* = 6.7 × 10^−14^) (Supplemental Table S3, Supplementary Figure S2). Notably, *GCNT4* is upregulated among smokers (Parker et al., 2017) (*p* = 9.8 × 10^−5^) while methylation of the DNAm site cg21158503 was decreased with smoking exposure (Joehanes et al., 2016) (*p* = 6.6 × 10^−6^).

**FIGURE 4:**
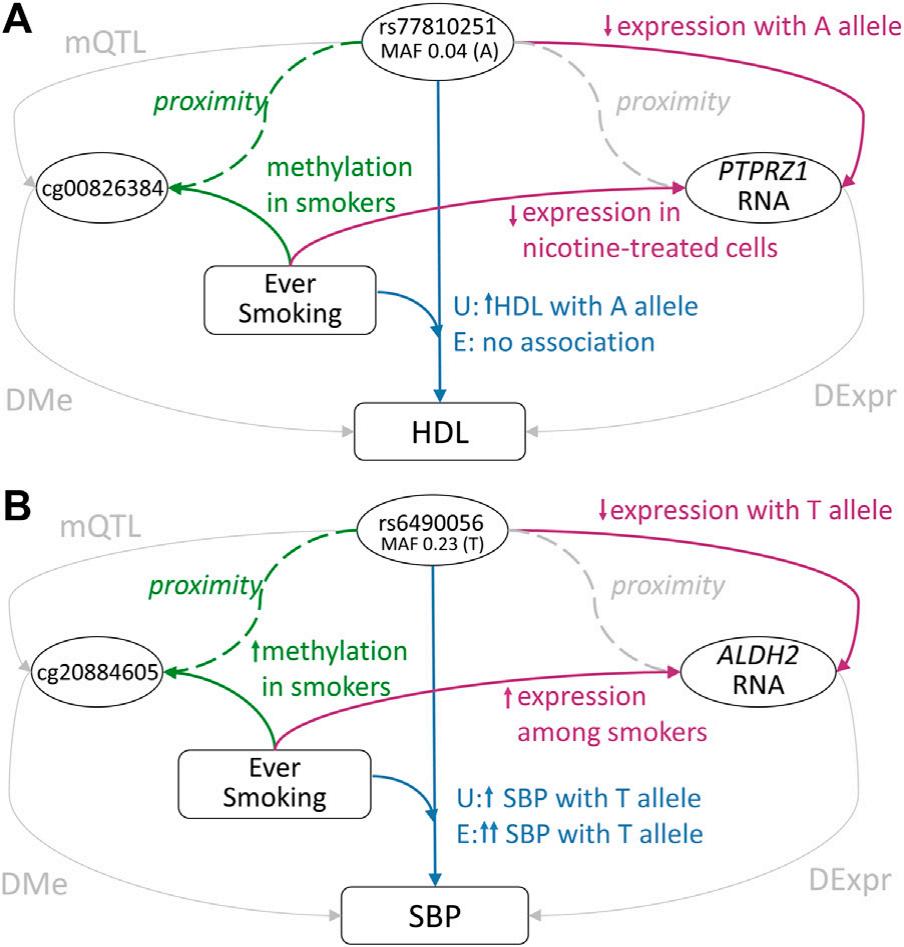
Summary of evidence for two GLI loci (see text for further details). Pathways without evidence for the association have been grayed out **(A)** *PTPRZ1* GLI Locus: the GLI variant rs77810251 in *PTPRZ1* is also an eQTL with downregulated expression in aortic tissue, and was in close proximity to the DNAm site cg00826384, which shows elevated methylation among smokers **(B)** *ALDH2* GLI Locus: the GLI variant rs6490056 in *ALDH2* is also an eQTL with downregulated expression in multiple tissues and was in close proximity to the DNAm site cg20884605, which shows elevated methylation among smokers. U: unexposed individuals, E: exposed individuals.

In the analysis of lipids and the ‘ever-smoking’ exposure in the African ancestry subset of cohorts, the association between the rs77810251 variant at the *PTPRZ1* locus and HDL levels was found to differ between exposure strata (Interaction *p* = 9.5 × 10^−7^), with a positive association of the minor A allele in the ‘never-smoking’ exposure group and no association among the ‘ever-smoking’ exposure group (Bentley et al., 2019) (Figure 4A, Supplemental Table S4, Supplementary Figure S3). rs77810251 is an eQTL for *PTPRZ1* in aorta tissue, with the A allele associated with decreased gene expression. A DNAm site within the locus, cg00826384, shows increased methylation among smokers (Joehanes et al., 2016). *PTPRZ1* was shown to be downregulated in nicotine-treated cells (Wang et al., 2011). These generate a potential hypothesis of the A allele of rs77810251 decreasing expression of *PTPRZ1*, which then causes an increase in HDL levels through unknown mechanisms. Smoking, which is associated with increased methylation of *PTPRZ1*, may perturb the *PTPRZ1*-HDL pathway, and abolished the association between rs77810251 and HDL. *PTPRZ1* is a protein tyrosine phosphatase receptor, which is constitutively active and is inactivated through binding with heparin-binding growth factors pleiotrophin and midkine. Its inactivation leads to increased tyrosine phosphorylation of target genes. This gene has a broad spectrum of substrates that may mediate multiple pathways (Shi et al., 2017). Of interest, both PTPRZ1 and LRP6, a member of the well-established lipids signaling family of LDL-receptor related proteins, are regulated through binding with midkine (Sakaguchi et al., 2003), suggesting a potential connection of this locus with a lipids pathway.

*SYN2* was prioritized from the PP and ‘current smoking’ GLI analysis among the African ancestry subset of cohorts. A rare T allele at a single variant in the locus, rs4135300, was associated with PP (*p* = 8.72 × 10^−7^) with a positive effect in the ‘non-current smoking’ exposure group and a negative effect in the ‘current smoking’ exposure group (Interaction P = 3 × 10^−7^; Supplemental Table S5). rs4135300 is an eQTL for *SYN2* in aorta tissue, with the T allele associated with decreased expression levels (Supplementary Figure S4). The DNAm site identified within the locus, cg10245988, has increased methylation on average among smokers (Joehanes et al., 2016). Expression of *SYN2* is increased in human neuroblastoma cells treated with nicotine. *SYN2* has previously been associated with schizophrenia (Mirnics et al., 2000).

The gene *ALDH2* was prioritized based on multiple analyses of smoking and blood pressure among the Asian ancestry subset of cohorts, specifically, the SBP with ‘current smoking’ (Figure 4B; Supplemental Table S7; Supplementary Figure S6), SBP with ‘ever smoking’ (Supplemental Table S8; Supplementary Figure S7), and MAP with ‘ever smoking’ Supplemental Table S6; Supplementary Figure S5) GLI analyses. As a representative example, the common T allele of rs6490056 was associated with increased SBP levels (*p* = 1.9 × 10^−10^) with a larger effect in the ‘current smoking’ exposure strata (Interaction *p* = 2.6 × 10^−5^). This variant and other associated variants in this locus are eQTLs for *ALDH2* in multiple tissues, including in the lungs and esophagus. *ALDH2* is a part of the major oxidative pathway for alcohol metabolism, and an Asian ancestry-specific variant (rs671) in this gene is well known for causing acetaldehyde accumulation with alcohol intake, leading to unpleasant side effects (Chen et al., 2014). Acetaldehyde and other toxic aldehydes are also components of tobacco smoke (Hoffman and Evans, 2013), and decreased ALDH2 activity leads to increased reactive aldehyde species and oxidative stress (Yasue et al., 2019). In individuals deficient in ALDH2 activity, smoking amplifies risk of oxidative stress-related conditions (Yasue et al., 2019). Importantly, oxidative stress is a known contributor to worsening blood pressure (Ahmad et al., 2017; Guzik and Touyz, 2017). Based on these data, it appears possible that rs6490056 reduces expression of *ALDH2*, raising oxidative stress, and causing a concomitant increase in blood pressure. Under the additional burden of oxidative stress introduced by smoking, an even stronger effect on blood pressure traits may be observed through this locus.

*TMEM116* is a transmembrane protein, expressed in nearly all measured tissues (Uhlén et al., 2015). Two variants at this locus (rs6490056, rs10849962) were associated in MAP with ‘ever smoking’ GLI analyses among the Asian ancestry subset of cohorts (Supplemental Table S9, Supplementary Figure S8). The common T allele of rs6490056 was associated with increased MAP measures (*p* = 5.7 × 10^−11^) with a larger effect in ‘ever smoking’ exposure group (Interaction *p* = 3.3 × 10^−5^). These variants were significant eQTLs with the T allele of rs6490056 associated with lower *TMEM116* expression levels in atrial and adipose tissues and the A allele of rs10849962 associated with higher *TMEM116* expression levels in a variety of tissues, including esophageal tissues, heart tissue, adipose, and whole blood. *TMEM116* is upregulated among smokers with corresponding demethylation of a nearby DNAm site, cg08528204 (Huan et al., 2016).

## 4 Discussion

Although gene × environment interactions are often cited as a potential source for “missing heritability” (Simon et al., 2016; Mayhew and Meyre, 2017), molecular evidence of these interactions remains limited. The establishment of CHARGE’s Gene Lifestyle Interactions Working Group resulted in a number of large-scale, trans-ancestry evaluations of gene × environment interaction effects (Feitosa et al., 2018; Sung et al., 2018; Bentley et al., 2019; de Vries et al., 2019; Sung et al., 2019). In this analysis, we leveraged publicly available epigenomic and transcriptomic summary association data to identify a subset of interactions from these analyses among which multiple forms of evidence point toward potential mechanistic explanations. We identified 833 genes for which evidence of a statistical interaction can be supported with functional data from at least one source, with 48 of these having multiple sources of functional support. We also observed an overrepresentation of prioritized interaction loci drawn from meta-analyses of African-ancestry populations. An overrepresentation of findings with the smoking vs. alcohol exposure may be a result of the greater amount of available omics data for smoking (Table 1).

A striking finding from this work is the large proportion of interactions that are observed among African ancestry meta-analyses compared to other ancestries or to Trans-ancestry analyses. This phenomenon was observed among both the full number of interactions identified (79.7%) as well as the prioritized loci (62.5%), and three of the five further prioritized loci were from African ancestry meta-analyses. This phenomenon reflects the underlying GLI association results from which our study draws. Notably, despite the shared methodology, this predominance of findings from African ancestry meta-analyses was evenly distributed among traits considered but was not evenly distributed among exposures, observed in studies of smoking with blood pressure and lipids, but not with alcohol exposure on either trait. Similarly, in these analyses, 26 of 30 of the prioritized loci based on meta-analyses of those of African ancestry were studies of the smoking exposure. A more complete discussion of these GLI association results are available in the primary publications for these projects. Briefly, some of the associations are for variants that are in higher frequency or present only among African ancestry populations, they are generally of low frequency (MAF 0.01–0.05) with high imputation scores, and with consistent associations across contributing African ancestry cohorts (Sung et al., 2018; Bentley et al., 2019; Sung et al., 2019; Laville et al., 2020). Of the 30 loci prioritized in this study based on African ancestry meta-analyses, the lead associated variant was African ancestry-specific (only present in 1 KG AFR populations) for only 2, while for most the lead variants were available in all ancestries, but not associated in the meta-analyses of other ancestries. These results suggest that the source of this observation relates to a smoking exposure-related difference by ancestry.

Although we did not have the data for a detailed evaluation of smoking patterns by ancestry in our studies, there are pronounced differences in smoking patterns across ancestry groups in the US (U.S. Department of Health and Human Services, 1998). Notably, there is a marked difference in the type of preferred cigarette, as shown in data from the National Survey on Drug Use and Health, which is designed to be representative of the US population: 88% of African Americans smokers vs. 26% of non-Hispanic white smokers used menthol cigarettes (Giovino et al., 2015). Menthol cigarettes are marketed more aggressively to African Americans (Lee et al., 2015; Mendez and Le, 2021). Additionally, some differences in preference may stem from variations in bitter taste perception by ancestry, with menthol cigarettes more palatable to those with stronger bitter taste perception as the menthol flavor additive masks the bitterness of nicotine. An African ancestry-specific genetic locus, *MRGPRX4*, associated with a five- to 8-fold increased odds of menthol cigarette smoking was recently identified (Kozlitina et al., 2019), although only a small minority of African Americans carry this variant.

Menthol cigarettes have long been targeted by the public health community based on evidence that they facilitate deeper smoke inhalation by decreasing nicotine-induced irritation (Ton et al., 2015; Mayhew and Meyre, 2017). This deeper inhalation may lead to a subsequent higher absorption of the myriad harmful components within cigarette smoke (U.S. Department of Health and Human Services, 1998; Ross et al., 2016). Consistent with this observation, ancestry differences in smoking-related metabolites and carcinogens have been reported (Pérez-Stable et al., 1998; Benowitz et al., 2011; Khariwala et al., 2014; Jain, 2015), and different levels of these compounds may underlie the observed differences by ancestry in genetic interactions upon smoking exposure. Additionally, there is some evidence for greater systemic oxidative stress (Il’yasova et al., 2012; Morris et al., 2012; Annor et al., 2017; Kim et al., 2020) and inflammation (Albert et al., 2004; Khera et al., 2005; Akinyemiju et al., 2019; Zahodne et al., 2019) among Americans of African vs. European ancestry. Exposure to cigarette smoke, a rich source of oxidants, on a background of elevated oxidative stress and inflammation may provoke a greater response among these individuals, manifesting as an interaction with smoking that differs by ancestry.

One key motivation for conducting analyses of gene × lifestyle interactions is the relative ease of practical translation, as results suggest a modifiable risk, i.e. individuals with a certain genotype might reduce exposures associated with exacerbated risk. In our efforts to map existing functional information to loci of interest, we identified several areas where improvements in available data might facilitate stronger inferences. For instance, there are limited data to evaluate differential gene expression by alcohol exposure, making it difficult to further investigate the loci identified in gene-alcohol interaction analyses. Additionally, more tissue-specific data would be useful. Specific tissue types are of greater interest for each phenotype (e.g. liver for lipids) and for each exposure (e.g. lung for smoking). Further, the reliance on whole blood, which is the most available in cohort studies, will limit our understanding of the underlying biology. Data linking methylation to gene expression is also limited. Although it was beyond the study design of this project, it would be useful to collect individual-level data to better elucidate these loci. Importantly, while some of the patterns observed in our data fit with expectations (e.g. the direction of RNA expression and DNA methylation for *GCNT4*, *PTPRZ1*, *TMEM116*), some did not, and individual-level data is needed to correctly model these associations.

A strength of these analyses is the use of the expansive genome-wide interaction results from large epidemiological meta-analyses. These data are drawn from discovery data on up to 133,805 individuals, important given the statistical power needed to detect gene-environment interaction effects. Additionally, the CHARGE Gene × Lifestyle Interactions Working Group went to great effort to include studies of diverse ancestries, such that relatively large proportions of historically underrepresented ancestry groups, such as African ancestry, were achieved. Given the preponderance of African ancestry-identified associations among our results, this inclusion was of key significance. This work could have been improved with omics data derived from individuals of diverse ancestries to explore associations that differed by ancestry. The exposure data considered in these analyses was represented using binary variables in order to maximize sample sizes for detection of interactions, although the true effects of exposures are certain to be more complex, with variations with timing and dose of exposure. Similarly, the exposures and phenotypes we selected for these analyses were relatively straightforward to measure, however, a wide range of exposures may be involved in gene-lifestyle interactions on a wide range of phenotypes, and it is unknown whether these findings are representative of gene-lifestyle interactions in general. Also, additional experimental data will be necessary to confirm biological pathways suggested by these findings in order to advance the evidence from this work towards clinical translation.

In summary, this work provides preliminary evidence from publicly available transcriptomics and epigenomics data to support the biological mechanisms underlying statistical gene × lifestyle interactions. These data suggest compelling evidence for how gene × lifestyle interactions may occur, motivating future studies that include individual-level epigenomic and transcriptomic data, other environmental exposures and outcomes, and more complex characterization of exposure.

## Data Availability

All of the data used in this work are publicly available. Both the original GWAS summary results and the re-processed statistics generated as part of this study are available *via* dbGaP (accession number phs000930).

## Abbreviations

*GLI*, Gene-lifestyle interaction; *LDL*, low density lipoprotein; *HDL*, high density lipoprotein; *TG*, triglycerides; *DBP*, diastolic blood pressure; *SBP*, systolic blood pressure; *MAP*, mean arterial pressure; *PP*, pulse pressure; *eQTL*, expression quantitative trait loci; *eQTM*, expression quantitative trait methylation; *mQTL*, methylation quantitative trait loci (SNPs whose genotype correlates with DNA methylation); *DNAm*, DNA methylation; *DMe*, differential methylation; *DExpr*, differential gene expression.
