# Supplementary material for: Multi-omics insights into the biological mechanisms underlying statistical gene-by-lifestyle interactions with smoking and alcohol consumption
Source: Front Genet. 2022 Dec 5;13:954713. doi: 10.3389/fgene.2022.954713 (PMC9760722; doi:10.3389/fgene.2022.954713)
Supplement: Supplementary file 1 [file DataSheet2.pdf]

Multi-omics insights into the biological mechanisms underlying statistical gene-by-lifestyle interactions with smoking and alcohol consumption

Timothy D. Majarian[1]*; Amy R. Bentley[2]*; Vincent LaVille[3]; Michael R. Brown[4]; Daniel I. Chasman[5]; Paul S. de Vries[4]; Mary F. Feitosa[6]; Nora Franceschini[7]; W. James Gauderman[8]; Casey Marchek[1,9]; Daniel Levy[10]; Alanna C. Morrison[4]; Michael Province[6]; Dabeeru C. Rao[11]; Karen Schwander[6,11]; Yun Ju Sung[11]; Charles N. Rotimi[2]; Hugues Aschard[3,12]; C. Charles Gu[11]; Alisa K. Manning[1,9,13] on behalf of the CHARGE Gene-Lifestyle Interactions Working Group

*co-first authors

[1]Program in Metabolism, Broad Institute of MIT and Harvard, Cambridge, MA, United States, [2]Center for Research on Genomics and Global Health, National Human Genome Research Institute, US National Institutes of Health, Bethesda, MD, United States, [3]Institut Pasteur, Université Paris Cité, Department of Computational Biology, F-75015 Paris, France, [4]Human Genetics Center, Department of Epidemiology, Human Genetics, and Environmental Sciences, School of Public Health, The University of Texas Health Science Center at Houston, Houston, TX, United States, [5]Division of Preventive Medicine, Brigham and Women's Hospital and Harvard Medical School, Boston, MA, United States, [6]Division of Statistical Genomics, Department of Genetics, Washington University School of Medicine, St. Louis, MO, United States, [7]Department of Epidemiology, Gillings School of Global Public Health, University of North Carolina at Chapel Hill, Chapel Hill, NC, United States, [8]Biostatistics, Department of Preventive Medicine, University of Southern California, Los Angeles, CA, United States, [9]Clinical and Translational Epidemiology Unit, Massachusetts General Hospital, Boston, MA, United States, [10]The Population Sciences Branch, National Heart, Lung, and Blood Institute, National Institutes of Health, Bethesda, MA, United States, [11]Division of Biostatistics, Washington University School of Medicine, St. Louis, MO, United States, [12]Program in Genetic Epidemiology and Statistical Genetics, Harvard T.H. Chan School of Public Health, Boston, MA, United States, [13]Department of Medicine and Harvard Medical School, Boston, MA, United States

Supplemental Tables

Supplemental Table 1: CHARGE genome-wide interaction study overview. Sample sizes represent combined Stage 1 (genome-wide) and Stage 2 (variants selected for follow-up).

		Sample Size (Proportion Exposed)				
**Outcome**	**Exposure**	**All**	**AA**	**ASA**	**EA**	**HA**
HDL	Current Drinking	390,558 (0.59)	25,464 (0.47)	120,881 (0.36)	226,505 (0.72)	17,708 (0.52)
	Drinking Habits	379,128 (0.43)	23,990 (0.20)	115,862 (0.31)	221,568 (0.52)	17,708 (0.26)
	Current Smoking	383,620 (0.20)	30,965 (0.22)	94,688 (0.20)	237,477 (0.20)	20,490 (0.17)
	Ever Smoking	381,875 (0.52)	29,365 (0.50)	94,688 (0.44)	237,332 (0.56)	20,490 (0.40)
LDL	Current Drinking	350,790 (0.56)	23,818 (0.47)	120,881 (0.36)	188,383 (0.71)	17,708 (0.52)
	Drinking Habits	337,804 (0.41)	22,344 (0.19)	114,012 (0.32)	183,740 (0.52)	17,708 (0.26)
	Current Smoking	310,093 (0.19)	28,672 (0.22)	61,466 (0.18)	199,958 (0.19)	19,997 (0.17)
	Ever Smoking	308,223 (0.51)	27,071 (0.49)	61,466 (0.40)	199,689 (0.56)	19,997 (0.40)

TG	Current Drinking	341,611 (0.57)	23,818 (0.47)	112,011 (0.38)	188,074 (0.71)	17,708 (0.52)
	Drinking Habits	335,494 (0.42)	22,344 (0.19)	112,011 (0.32)	183,431 (0.52)	17,708 (0.26)
	Current Smoking	352,138 (0.20)	28,859 (0.22)	116,570 (0.20)	186,196 (0.19)	20,513 (0.17)
	Ever Smoking	349,979 (0.52)	27,258 (0.49)	116,570 (0.48)	185,638 (0.56)	20,513 (0.40)

SBP	Current Drinking	569,702 (0.67)	26,458 (0.47)	153,391 (0.37)	372,420 (0.81)	17,433 (0.51)
	Drinking Habits	241,295 (0.36)	10,627 (0.15)	54,212 (0.53)	173,489 (0.32)	2,967 (0.15)
	Current Smoking	600,210 (0.18)	34,901 (0.22)	162,370 (0.20)	380,612 (0.17)	22,327 (0.17)
	Ever Smoking	582,290 (0.48)	31,994 (0.49)	162,370 (0.46)	365,592 (0.50)	22,334 (0.38)
DBP	Current Drinking	569,748 (0.67)	26,457 (0.47)	153,389 (0.37)	372,475 (0.81)	17,427 (0.51)
	Drinking Habits	241,347 (0.36)	10,627 (0.15)	54,212 (0.53)	173,541 (0.32)	2,967 (0.15)
	Current Smoking	600,210 (0.18)	34,901 (0.22)	162,370 (0.20)	380,612 (0.17)	22,327 (0.17)
	Ever Smoking	582,290 (0.48)	31,994 (0.49)	162,370 (0.46)	365,592 (0.50)	22,334 (0.38)
MAP	Current Drinking	569,702 (0.67)	26,458 (0.47)	153,391 (0.37)	372,420 (0.81)	17,433 (0.51)
	Drinking Habits	241,295 (0.36)	10,627 (0.15)	54,212 (0.53)	173,489 (0.32)	2,967 (0.15)
	Current Smoking	600,210 (0.18)	34,901 (0.22)	162,370 (0.20)	380,612 (0.17)	22,327 (0.17)
	Ever Smoking	582,290 (0.48)	31,994 (0.49)	162,370 (0.46)	365,592 (0.50)	22,334 (0.38)
PP	Current Drinking	569,702 (0.67)	26,458 (0.47)	153,391 (0.37)	372,420 (0.81)	17,433 (0.51)
	Drinking Habits	241,295 (0.36)	10,627 (0.15)	54,212 (0.53)	173,489 (0.32)	2,967 (0.15)
	Current Smoking	600,210 (0.18)	34,901 (0.22)	162,370 (0.20)	380,612 (0.17)	22,327 (0.17)
	Ever Smoking	582,290 (0.48)	31,994 (0.49)	162,370 (0.46)	365,592 (0.50)	22,334 (0.38)

Supplemental Table 2. Prioritized loci with full eQTL information

Exposure	Gene Target	eQTL Tissue(s)	IntP < 5e-5
African Ancestry HDL			
Curr Smk	POLK	Brain Cerebellum, Heart Left Ventricle, Nerve Tibial, Pancreas, Spleen, Thyroid, Brain Cerebellar Hemisphere	5
Curr Smk	*GCNT4*[1]	Artery Aorta, Testis	4
Curr Smk	*KLK8*	Skin Sun Exposed Lower leg	4
Curr Smk	*ANGPT1*	Thyroid	3
Curr Smk	*CARM1*[2]	Muscle Skeletal	1
Curr Smk	*LIPC*	Liver, Pancreas	1
Curr Smk	*YIPF2*[2]	Lung	1
Ev Smk	*PTPRZ1*[1,2]	Artery Aorta	21
Ev Smk	*DNAH7*	Adipose Subcutaneous, Adipose Visceral Omentum, Artery Tibial, Brain Caudate basal ganglia, Brain Cortex, Brain Nucleus accumbens basal ganglia, Brain Putamen basal ganglia, Breast Mammary Tissue, Cells Transformed fibroblasts, Esophagus Muscularis, Nerve Tibial, Ovary, Pancreas, Skin Not Sun Exposed Suprapubic, Skin Sun Exposed Lower leg, Stomach, Thyroid	1
African Ancestry LDL			
Ev Smk	*CRYGN*	Colon Sigmoid, Nerve Tibial	1
Ev Smk	*FRK*	Muscle Skeletal	1
Ev Smk	*WDR86*	Nerve Tibial	1
African Ancestry TG			
Curr Drnk	*BAHD1*	Colon Sigmoid, Esophagus Muscularis, Nerve Tibial, Pancreas, Stomach, Thyroid	2
Curr Drnk	*DNAJC17*	Esophagus Mucosa	2
Ev Smk	*TRIO*	Ovary	1

African Ancestry DBP			
Ev Smk	*FNTB*	Muscle Skeletal, Skin Sun Exposed Lower leg	3
Ev Smk	*RAB15*	Adipose Subcutaneous, Cells Transformed fibroblasts, Esophagus Muscularis, Nerve Tibial, Testis, Thyroid	3
African Ancestry MAP			
Curr Smk	*CRTAC1*	Adipose Visceral Omentum, Artery Tibial	1
Curr Smk	*SULT4A1*	Colon Sigmoid	1
Ev Smk	*FNTB*	Muscle Skeletal, Skin Sun Exposed Lower leg	3
Ev Smk	*RAB15*	Adipose Subcutaneous, Cells Transformed fibroblasts, Esophagus Muscularis, Nerve Tibial, Testis, Thyroid	3
Ev Smk	*KCNK13*	Brain Hypothalamus	1
African Ancestry PP			
Curr Drnk	*MPI*	Adipose Subcutaneous, Adipose Visceral Omentum, Artery Aorta, Artery Tibial, Brain Anterior cingulate cortex BA24, Brain Caudate basal ganglia, Brain Cerebellum, Brain Cortex, Brain Nucleus accumbens basal ganglia, Cells Transformed fibroblasts, Esophagus Mucosa, Esophagus Muscularis, Lung, Nerve Tibial, Skin Not Sun Exposed Suprapubic, Skin Sun Exposed Lower leg, Spleen, Testis, Thyroid, Whole Blood	4
Curr Drnk	*SCAMP2*	Esophagus Mucosa, Pituitary, Skin Sun Exposed Lower leg, Testis, Whole Blood	4
Curr Smk	*SYN2*[1]	Artery Aorta	1
Ev Smk	*JMJD4*	Nerve Tibial	2
African Ancestry SBP			
Curr Smk	*CRTAC1*	Adipose Visceral Omentum, Artery Tibial	1
Ev Smk	*FNTB*	Muscle Skeletal, Skin Sun Exposed Lower leg	2
Ev Smk	*RAB15*	Adipose Subcutaneous, Cells Transformed fibroblasts, Esophagus Muscularis, Nerve Tibial, Testis, Thyroid	2
Ev Smk	*JMJD4*	Nerve Tibial	1
Asian Ancestry HDL			

Ev Smk	*KCTD10*[2]	Artery Tibial, Skin Sun Exposed Lower leg	1
Ev Smk	*MYO1H*[2]	Lung	1
Ev Smk	*UBE3B*[2]	Colon Transverse, Skin Sun Exposed Lower leg	1
Asian Ancestry LDL			
Drnk habits	*CPNE2*	Cells Transformed fibroblasts	3
Ev Smk	*KANK2*[2]	Adipose Subcutaneous, Cells Transformed fibroblasts, Esophagus Muscularis, Lung, Nerve Tibial, Skin Sun Exposed Lower leg, Thyroid	2
Asian Ancestry TG			
Drnk habits	*LIPC*[2]	Thyroid	1
Asian Ancestry MAP			
Ev Smk	*ALDH2*[1,2]	Esophagus Mucosa, Skin Sun Exposed Lower leg, Lung, Nerve Tibial, Skin Not Sun Exposed Suprapubic, Thyroid	2
Ev Smk	*TMEM116*[1,2]	Adipose Visceral Omentum, Artery Tibial, Brain Frontal Cortex BA9, Esophagus Gastroesophageal Junction, Esophagus Mucosa, Esophagus Muscularis, Heart Atrial Appendage, Muscle Skeletal, Whole Blood-exp	1
Asian Ancestry SBP			
Curr Smk	*ALDH2*[1,2]	Esophagus Mucosa, Skin Sun Exposed Lower leg, Brain Cortex, Thyroid, Skin Not Sun Exposed Suprapubic	3
Ev Smk	*ALDH2*[1,2]	Esophagus Mucosa, Skin Sun Exposed Lower leg	1
European Ancestry LDL			
Drnk habits	*APOC1*[2]	Esophagus Mucosa	1
European Ancestry TG			
Drnk habits	*KRTCAP3*[2]	Adrenal Gland, Lung, Muscle Skeletal, Spleen, Thyroid, Whole Blood, Brain Caudate basal ganglia, Brain Cortex, Esophagus Mucosa, Skin Sun Exposed Lower leg	2
Drnk habits	*PPM1G*[2]	Esophagus Muscularis, Muscle Skeletal, Adipose Subcutaneous, Skin Sun Exposed Lower leg, Stomach, Thyroid	2

Hispanic Ancestry PP			
Curr Smk	*STIM1*	Lung, Thyroid, Whole Blood	2
Trans Ancestry LDL			
Ev Smk	*CRYGN*	Colon Sigmoid, Nerve Tibial	1
Ev Smk	*WDR86*	Nerve Tibial	1
Trans Ancestry TG			
Drnk habits	*KRTCAP3*[2]	Adrenal Gland, Lung, Muscle Skeletal, Spleen, Thyroid, Whole Blood	1
Drnk habits	*PPM1G*[2]	Esophagus Muscularis, Muscle Skeletal	1

[1]Differential Expression by Exposure; [2]Loci with 2df GLI association

Supplemental Table 3: *GNCT4* × Current Smoking and HDL (African Ancestry)

rsID	2df P-value	SNP Main Effect	Interaction P-value	Interaction Effect	eQTL Tissue	eQTL Effect	eQTL P-value
rs11957913	0.0002049	0.003585	4.02E-05	-0.0239	Artery_Aorta	-	7.95E-06
rs11957913	0.0002049	0.003585	4.02E-05	-0.0239	Testis	+	1.09E-13
rs6886841	0.0003427	0.002521	3.45E-05	-0.024	Artery_Aorta	-	2.96E-06
rs6886841	0.0003427	0.002521	3.45E-05	-0.024	Testis	+	2.45E-13
rs3761743	0.0003735	0.002179	1.55E-05	-0.0249	Artery_Aorta	-	3.52E-06
rs3761743	0.0003735	0.002179	1.55E-05	-0.0249	Testis	+	6.72E-14
rs7737959	0.0006878	0.002938	3.65E-05	-0.0241	Artery_Aorta	-	4.46E-06
rs7737959	0.0006878	0.002938	3.65E-05	-0.0241	Testis	+	1.01E-13
rs7356637	0.0009189	0.003	4.88E-05	-0.0237	Artery_Aorta	-	8.40E-06
rs7356637	0.0009189	0.003	4.88E-05	-0.0237	Testis	+	1.28E-13

Site name	P-value	Effect	Effect type	Notes
cg21158502	6.62E-06	-0.006	Smoking on methylation (Difference in mean methylation)	ARIC, FHS Offspring, KORA F4, GOLDN, LBC 1921, LBC 1936, NAS, Rotterdam, Inchianti, GTP, CHS European Ancestry (EA), CHS African Ancestry (AA), GENOA, EPIC Norfolk, EPIC, and MESA [Joehanes]
GCNT4	9.88E-05	0.105	Smoking on expression (Fold change)	COPD Gene: self-identified Non- Hispanic Whites and African Americans between the ages of 45 and 80 years with a minimum of 10 pack-years life- time smoking history [Parker]

Supplemental Table 4: *PTPRZ1* × Ever Smoked and HDL (African Ancestry)

rsID	2df P-value	SNP Main Effect	Interaction P-value	Interaction Effect	eQTL Tissue	eQTL Effect	eQTL P-value
rs77810251	1.24E-09	0.05244	9.55E-07	-0.0589	Artery_Aorta	-	1.47E-08
rs6966138	5.53E-09	0.04933	2.42E-06	-0.0555	Artery_Aorta	-	1.47E-08
rs138601001	5.76E-09	0.04942	2.49E-06	-0.0554	Artery_Aorta	-	1.57E-08
rs3757551	8.63E-09	0.04895	3.02E-06	-0.055	Artery_Aorta	-	1.47E-08
rs6965735	9.41E-09	-0.04875	3.30E-06	0.0548	Artery_Aorta	+	1.82E-08
rs2109767	1.21E-08	-0.04739	2.79E-06	0.054	Artery_Aorta	+	1.01E-08
rs74755112	1.25E-08	0.04734	1.52E-06	-0.0556	Artery_Aorta	-	1.47E-08
rs75052642	1.48E-08	0.04815	4.52E-06	-0.054	Artery_Aorta	-	1.47E-08
rs740965	1.52E-08	-0.0482	4.54E-06	0.054	Artery_Aorta	+	1.01E-08
rs73229136	7.22E-07	0.02479	2.94E-06	-0.0343	Artery_Aorta	-	2.59E-07
rs3801377	1.49E-06	-0.02648	4.44E-06	0.0363	Artery_Aorta	+	1.92E-07
rs1916885	1.84E-06	-0.02333	3.15E-06	0.034	Artery_Aorta	+	2.59E-07
rs12669706	2.65E-06	0.02595	5.66E-06	-0.0358	Artery_Aorta	-	1.92E-07
rs56265902	2.69E-06	0.02611	5.31E-06	-0.0359	Artery_Aorta	-	3.78E-07
rs12672519	3.79E-06	0.02559	9.72E-06	-0.0345	Artery_Aorta	-	1.63E-07
rs12667535	5.35E-06	0.02473	6.61E-06	-0.0353	Artery_Aorta	-	1.92E-07
rs3801374	6.28E-06	0.02463	7.96E-06	-0.035	Artery_Aorta	-	1.92E-07
rs6971645	6.80E-06	-0.02413	5.18E-06	0.0353	Artery_Aorta	+	1.92E-07
rs3801373	7.01E-06	0.02447	8.03E-06	-0.0349	Artery_Aorta	-	2.38E-07
rs2024251	7.03E-06	0.02451	8.97E-06	-0.0348	Artery_Aorta	-	1.92E-07
rs9641678	1.24E-05	-0.02355	9.09E-06	0.0346	Artery_Aorta	+	1.92E-07

Site name	Pvalue	Effect	Effect type	Notes
cg00826384	0.0011	1.90E-03	Smoking on methylation (Difference in mean methylation)	ARIC, FHS Offspring, KORA F4, GOLDN, LBC 1921, LBC 1936, NAS, Rotterdam, Inchianti, GTP, CHS European Ancestry (EA), CHS African Ancestry (AA), GENOA, EPIC Norfolk, EPIC, and MESA [Joehanes]
PTPRZ1	0.041	0.9	Smoking on expression (fold change)	human neuroblastoma SH-SY5Y cells [Wang]

Supplemental Table 5: *SYN2* × Current Smoking on PP (African Ancestry)

rsID	2df P-value	SNP Main Effect	Interaction P-value	Interaction Effect	eQTL Tissue	eQTL Effect	eQTL P-value
rs4135300	8.72E-07	2.259	3.02E-07	-9.8896	Artery_Aorta	-	2.19E-05
**Site name**	**P-value**	**Effect**	**Effect type**		**Notes**		
cg10245988	9.00E-04	3.40E-03	Smoking on methylation (Difference in mean methylation)		ARIC, FHS Offspring, KORA F4, GOLDN, LBC 1921, LBC 1936, NAS, Rotterdam, Inchianti, GTP, CHS European Ancestry (EA), CHS African Ancestry (AA), GENOA, EPIC Norfolk, EPIC, and MESA [Joehanes]		
SYN2	0.014	1.25	Smoking on expression (fold change)		human neuroblastoma SH-SY5Y cells [Wang]		

Supplemental Table 6: *ALDH2* × Ever Smoked and MAP (Asian Ancestry)

rsID	2df P-value	SNP Main Effect	Interaction P-value	Interaction Effect	eQTL Tissue	eQTL Effect	eQTL P-value
rs6490056	5.74E-11	0.1013	3.31E-05	0.5215	Skin Sun Exposed Lower leg	-	8.53E-08
rs6490056	5.74E-11	0.1013	3.31E-05	0.5215	Esophagus Mucosa	-	2.42E-05
rs10849962	2.62E-09	0.04002	4.69E-05	0.5046	Lung	+	5.76E-07
rs10849962	2.62E-09	0.04002	4.69E-05	0.5046	Nerve Tibial	+	5.09E-07
rs10849962	2.62E-09	0.04002	4.69E-05	0.5046	Skin Not Sun Exposed Suprapubic	+	3.48E-08
rs10849962	2.62E-09	0.04002	4.69E-05	0.5046	Esophagus Mucosa	+	1.54E-10
rs10849962	2.62E-09	0.04002	4.69E-05	0.5046	Skin Sun Exposed Lower leg	+	8.45E-12
rs10849962	2.62E-09	0.04002	4.69E-05	0.5046	Thyroid	+	2.93E-06
rs6490056	5.74E-11	0.1013	3.31E-05	0.5215	Skin Sun Exposed Lower leg	-	8.53E-08
**Site name**	**P-value**	**Effect**	**Effect type**		**Notes**		
cg20884605	4.90E-07	4.30E-03	Smoking on methylation (Difference in mean methylation)		ARIC, FHS Offspring, KORA F4, GOLDN, LBC 1921, LBC 1936, NAS, Rotterdam, Inchianti, GTP, CHS European Ancestry (EA), CHS African Ancestry (AA), GENOA, EPIC Norfolk, EPIC, and MESA [Joehanes]		
ALDH2	9.68E-09	8.45E-02	Smoking on expression (fold change)		Framingham Heart Study (FHS), the Rotterdam Study (RS), the Cooperative Health Research in the Region of Augsburg (KORA F4) Study, the InCHIANTI Study, the Study of Health in Pomerania (SHIP-TREND), and the Estonian Biobank (EGCUT) [Huan]		

Supplemental Table 7: *ALDH2* × Current Smoking and SBP (Asian Ancestry)

rsID	2df P-value	SNP Main Effect	Interaction P-value	Interaction Effect	eQTL Tissue	eQTL Effect	eQTL P-value
rs10774621	9.69E-13	-0.3637	3.39E-05	-0.8977	Esophagus Mucosa	+	2.04E-05
rs10774621	9.69E-13	-0.3637	3.39E-05	-0.8977	Skin Sun Exposed Lower leg	+	2.06E-07
rs10774621	9.69E-13	-0.3637	3.39E-05	-0.8977	Brain Cortex	+	4.45E-06
rs10774621	9.69E-13	-0.3637	3.39E-05	-0.8977	Thyroid	+	1.04E-06
rs73412370	1.23E-12	0.348	3.76E-05	0.8617	Brain Cortex	-	3.78E-06
rs73412370	1.23E-12	0.348	3.76E-05	0.8617	Esophagus Mucosa	-	3.27E-05
rs73412370	1.23E-12	0.348	3.76E-05	0.8617	Skin Not Sun Exposed Suprapubic	-	8.35E-06
rs73412370	1.23E-12	0.348	3.76E-05	0.8617	Skin Sun Exposed Lower leg	-	2.36E-07
rs73412370	1.23E-12	0.348	3.76E-05	0.8617	Thyroid	-	2.82E-08
rs6490056	1.99E-10	0.2777	2.75E-05	0.9462	Esophagus Mucosa	-	2.42E-05
rs6490056	1.99E-10	0.2777	2.75E-05	0.9462	Skin Sun Exposed Lower leg	-	8.53E-08
rs10774621	9.69E-13	-0.3637	3.39E-05	-0.8977	Esophagus Mucosa	+	2.04E-05

Site name	P-value	Effect	Effect type	Notes
cg20884605	4.90E-07	4.30E-03	Smoking on methylation (Difference in mean methylation)	ARIC, FHS Offspring, KORA F4, GOLDN, LBC 1921, LBC 1936, NAS, Rotterdam, Inchianti, GTP, CHS European Ancestry (EA), CHS African Ancestry (AA), GENOA, EPIC Norfolk, EPIC, and MESA [Joehanes]
ALDH2	9.68E-09	8.45E-02	Smoking on expression (fold change)	Framingham Heart Study (FHS), the Rotterdam Study (RS), the Cooperative Health Research in the Region of Augsburg (KORA F4) Study, the InCHIANTI Study, the Study of Health in Pomerania (SHIP-TREND), and the Estonian Biobank (EGCUT) [Huan]

Supplemental Table 8: *ALDH2* × Ever Smoked and SBP (Asian Ancestry)

rsID	2df P-value	SNP Main Effect	Interaction P-value	Interaction Effect	eQTL Tissue	eQTL Effect	eQTL P-value
rs6490056	3.42E-10	0.1131	3.78E-05	0.7353	Esophagus Mucosa	-	2.42E-05
rs6490056	3.42E-10	0.1131	3.78E-05	0.7353	Skin Sun Exposed Lower leg	-	8.53E-08
**Site name**	**P-value**	**Effect**	**Effect type**		**Notes**		
cg20884605	4.90E-07	4.30E-03	Smoking on methylation (Difference in mean methylation)		ARIC, FHS Offspring, KORA F4, GOLDN, LBC 1921, LBC 1936, NAS, Rotterdam, Inchianti, GTP, CHS European Ancestry (EA), CHS African Ancestry (AA), GENOA, EPIC Norfolk, EPIC, and MESA [Joehanes]		
ALDH2	9.68E-09	8.45E-02	Smoking on expression (fold change)		Framingham Heart Study (FHS), the Rotterdam Study (RS), the Cooperative Health Research in the Region of Augsburg (KORA F4) Study, the InCHIANTI Study, the Study of Health in Pomerania (SHIP-TREND), and the Estonian Biobank (EGCUT) [Huan]		

Supplemental Table 9: *TMEM116* × Ever Smoked and MAP (Asian Ancestry)

rsID	2df Pvalue	SNP main Effect	Interaction Pvalue	Interaction Effect	eQTL Tissue	eQTL Effect	eQTL Pvalue
rs6490056	5.74E-11	0.1013	3.31E-05	0.5215	Heart Atrial Appendage	+	8.59E-06
rs6490056	5.74E-11	0.1013	3.31E-05	0.5215	Adipose Visceral Omentum	+	5.88E-06
rs10849962	2.62E-09	0.04002	4.69E-05	0.5046	Esophagus Gastroesophageal Junction	-	2.83E-06
rs10849962	2.62E-09	0.04002	4.69E-05	0.5046	Adipose Visceral Omentum	-	7.58E-07
rs10849962	2.62E-09	0.04002	4.69E-05	0.5046	Artery Tibial	-	2.50E-05
rs10849962	2.62E-09	0.04002	4.69E-05	0.5046	Brain Frontal Cortex BA9	-	3.62E-05
rs10849962	2.62E-09	0.04002	4.69E-05	0.5046	Heart Atrial Appendage	-	2.03E-07
rs10849962	2.62E-09	0.04002	4.69E-05	0.5046	Esophagus Mucosa	-	6.11E-05
rs10849962	2.62E-09	0.04002	4.69E-05	0.5046	Esophagus Muscularis	-	6.87E-06
rs10849962	2.62E-09	0.04002	4.69E-05	0.5046	Muscle Skeletal	-	1.25E-06
rs10849962	2.62E-09	0.04002	4.69E-05	0.5046	Whole Blood	-	2.98E-05

Site name	P-value	Effect	Effect type	Notes
cg08528204	2.07E-08	0.03	Smoking on methylation (Difference in mean methylation)	Family and Community Health Study (FACHS) (African American females from Iowa and Georgia, n=111) [Huan]
TMEM116	8.56E-04	3.76E-02	Smoking on expression (fold change)	Framingham Heart Study (FHS), the Rotterdam Study (RS), the Cooperative Health Research in the Region of Augsburg (KORA F4) Study, the InCHIANTI Study, the Study of Health in Pomerania (SHIP-TREND), and the Estonian Biobank (EGCUT) [Huan]
