# Supplementary material for: Multi-omics insights into the biological mechanisms underlying statistical gene-by-lifestyle interactions with smoking and alcohol consumption
Source: Front Genet. 2022 Dec 5;13:954713. doi: 10.3389/fgene.2022.954713 (PMC9760722; doi:10.3389/fgene.2022.954713)
Supplement: Supplementary file 2 [file DataSheet1.pdf]

Multi-omics insights into the biological mechanisms underlying statistical gene-by-lifestyle interactions with smoking and alcohol consumption

Timothy D. Majarian[1]*; Amy R. Bentley[2]*; Vincent LaVille[3]; Michael R. Brown[4]; Daniel I. Chasman[5]; Paul S. de Vries[4]; Mary F. Feitosa[6]; Nora Franceschini[7]; W. James Gauderman[8]; Casey Marchek[1,9]; Daniel Levy[10]; Alanna C. Morrison[4]; Michael Province[6]; Dabeeru C. Rao[11]; Karen Schwander[6,11]; Yun Ju Sung[11]; Charles N. Rotimi[2]; Hugues Aschard[3,12]; C. Charles Gu[11]; Alisa K. Manning[1,9,13] on behalf of the CHARGE Gene-Lifestyle Interactions Working Group
*co-first authors
[1]Program in Metabolism, Broad Institute of MIT and Harvard, Cambridge, MA, United States, [2]Center for Research on Genomics and Global Health, National Human Genome Research Institute, US National Institutes of Health, Bethesda, MD, United States, [3]Institut Pasteur, Université Paris Cité, Department of Computational Biology, F-75015 Paris, France, [4]Human Genetics Center, Department of Epidemiology, Human Genetics, and Environmental Sciences, School of Public Health, The University of Texas Health Science Center at Houston, Houston, TX, United States, [5]Division of Preventive Medicine, Brigham and Women's Hospital and Harvard Medical School, Boston, MA, United States, [6]Division of Statistical Genomics, Department of Genetics, Washington University School of Medicine, St. Louis, MO, United States, [7]Department of Epidemiology, Gillings School of Global Public Health, University of North Carolina at Chapel Hill, Chapel Hill, NC, United States, [8]Biostatistics, Department of Preventive Medicine, University of Southern California, Los Angeles, CA, United States, [9]Clinical and Translational Epidemiology Unit, Massachusetts General Hospital, Boston, MA, United States, [10]The Population Sciences Branch, National Heart, Lung, and Blood Institute, National Institutes of Health, Bethesda, MA, United States, [11]Division of Biostatistics, Washington University School of Medicine, St. Louis, MO, United States, [12]Program in Genetic Epidemiology and Statistical Genetics, Harvard T.H. Chan School of Public Health, Boston, MA, United States, [13]Department of Medicine and Harvard Medical School, Boston, MA, United States

Supplemental Figures

Supplemental Figure 1. Manhattan plots of GLI association results with lipids (top) and blood pressure (bottom) in (A) trans-ancestry meta analyses, (B) African Ancestry subgroup, (C) Asian Ancestry subgroup, (D) European Ancestry subgroup, and (E) Hispanic Ancestry subgroup analyses.

A.

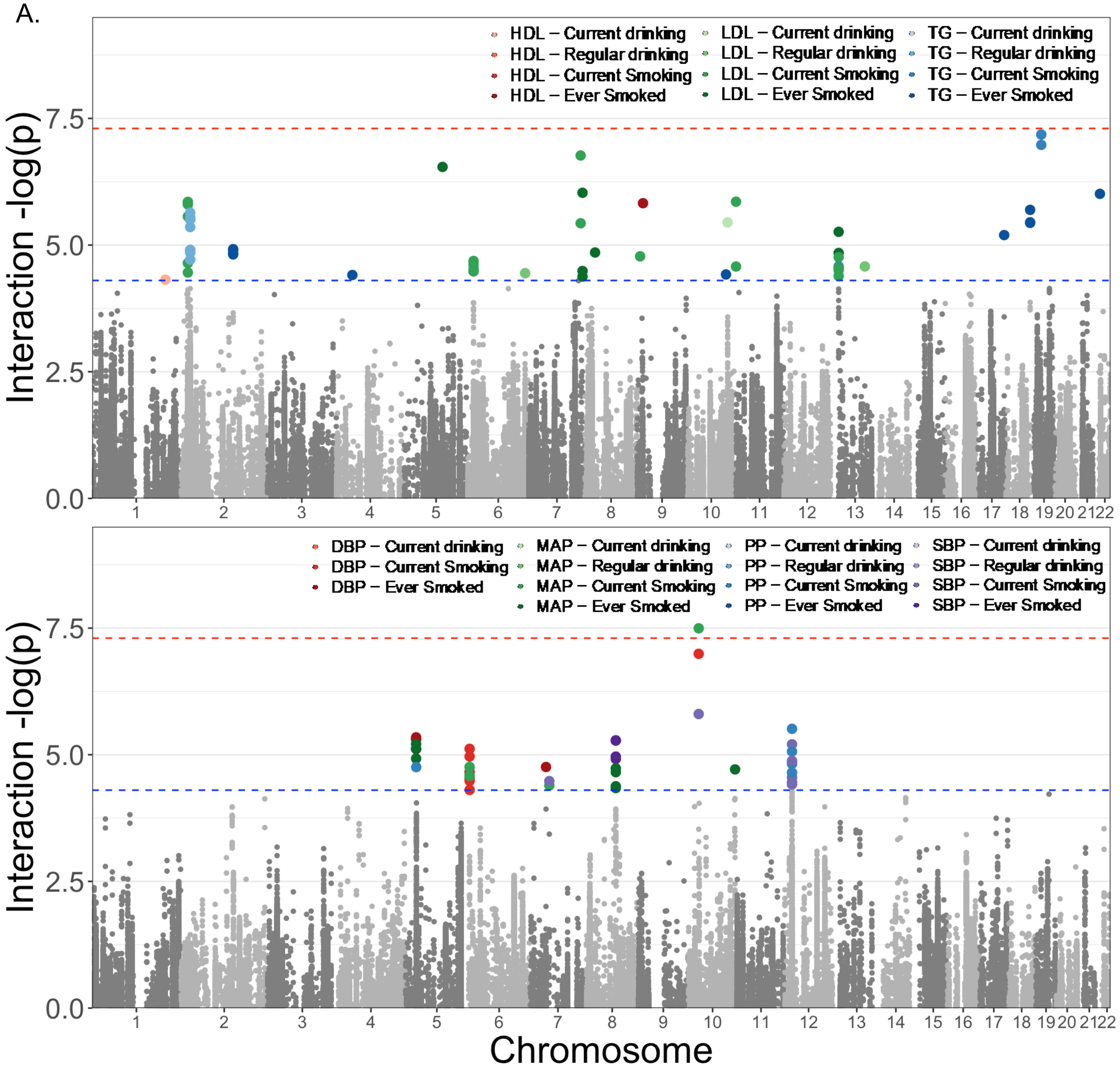

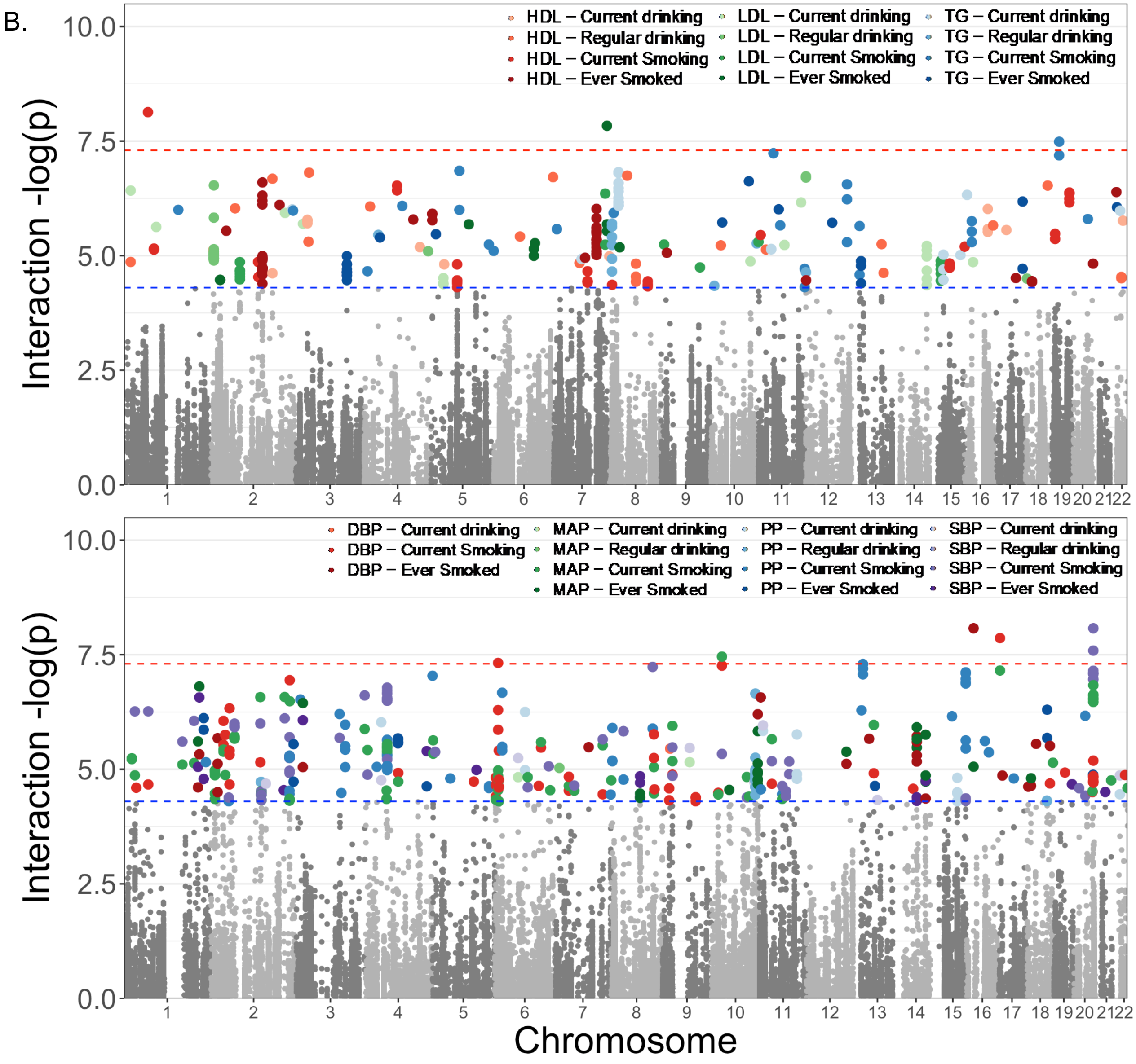
B.
HDL – Current drinking
HDL – Regular drinking
HDL – Current Smoking
HDL – Ever Smoked
LDL – Current drinking
LDL – Regular drinking
LDL – Current Smoking
LDL – Ever Smoked
TG – Current drinking
TG – Regular drinking
TG – Current Smoking
TG – Ever Smoked
DBP – Current drinking
DBP – Current Smoking
DBP – Ever Smoked
MAP – Current drinking
MAP – Regular drinking
MAP – Current Smoking
MAP – Ever Smoked
PP – Current drinking
PP – Regular drinking
PP – Current Smoking
PP – Ever Smoked
SBP – Current drinking
SBP – Regular drinking
SBP – Current Smoking
SBP – Ever Smoked
Interaction -log(p)
0.0
2.5
5.0
7.5
10.0
1 2 3 4 5 6 7 8 9 10 11 12 13 14 15 16 17 18 19 20 21 22
Chromosome

C.

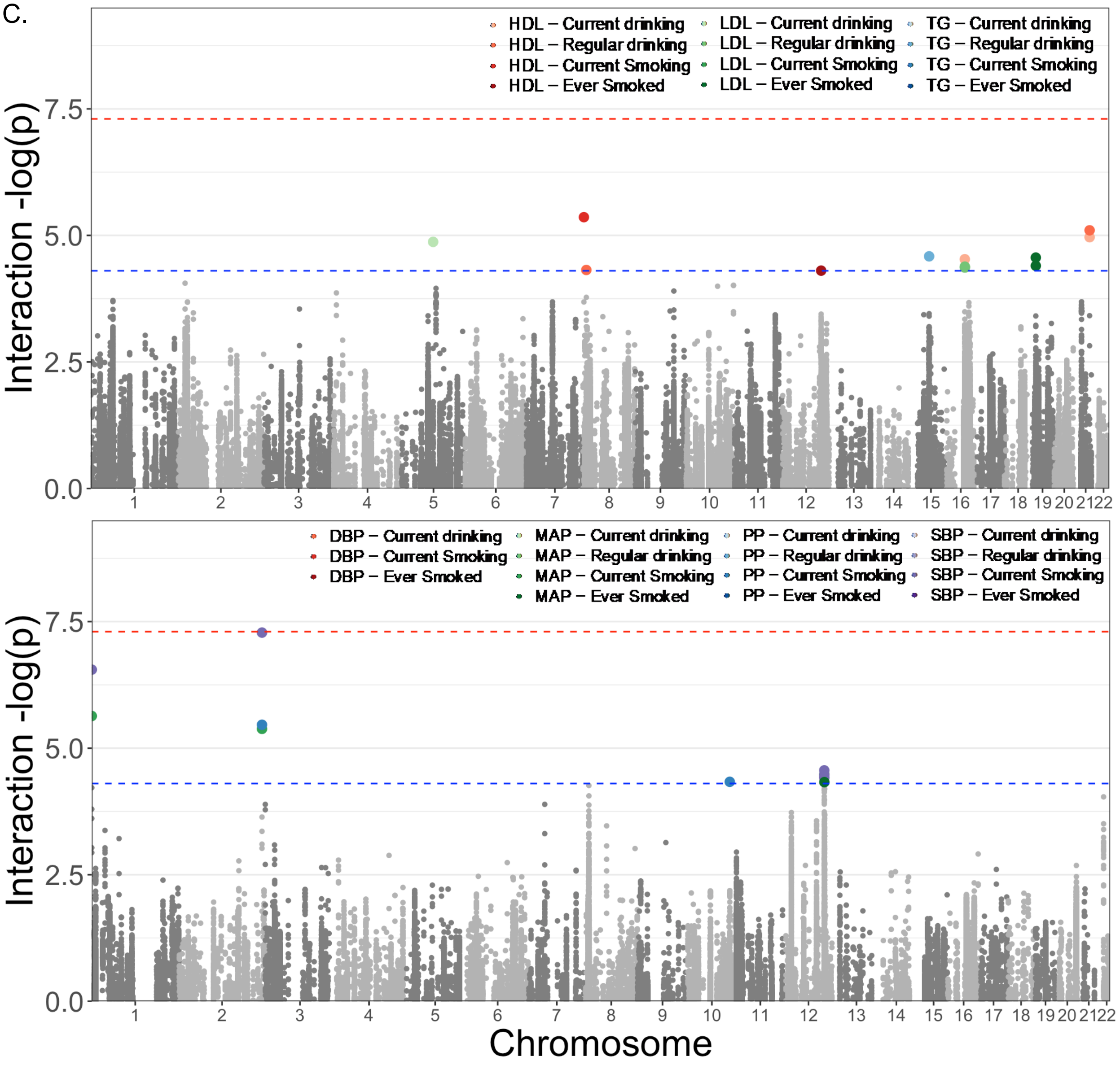
HDL – Current drinking
HDL – Regular drinking
HDL – Current Smoking
HDL – Ever Smoked
LDL – Current drinking
LDL – Regular drinking
LDL – Current Smoking
LDL – Ever Smoked
TG – Current drinking
TG – Regular drinking
TG – Current Smoking
TG – Ever Smoked
Interaction -log(p)
7.5
5.0
2.5
0.0
1
2
3
4
5
6
7
8
9
10
11
12
13
14
15
16
17
18
19
20
21
22
DBP – Current drinking
DBP – Current Smoking
DBP – Ever Smoked
MAP – Current drinking
MAP – Regular drinking
MAP – Current Smoking
MAP – Ever Smoked
PP – Current drinking
PP – Regular drinking
PP – Current Smoking
PP – Ever Smoked
SBP – Current drinking
SBP – Regular drinking
SBP – Current Smoking
SBP – Ever Smoked
Interaction -log(p)
Chromosome

D.

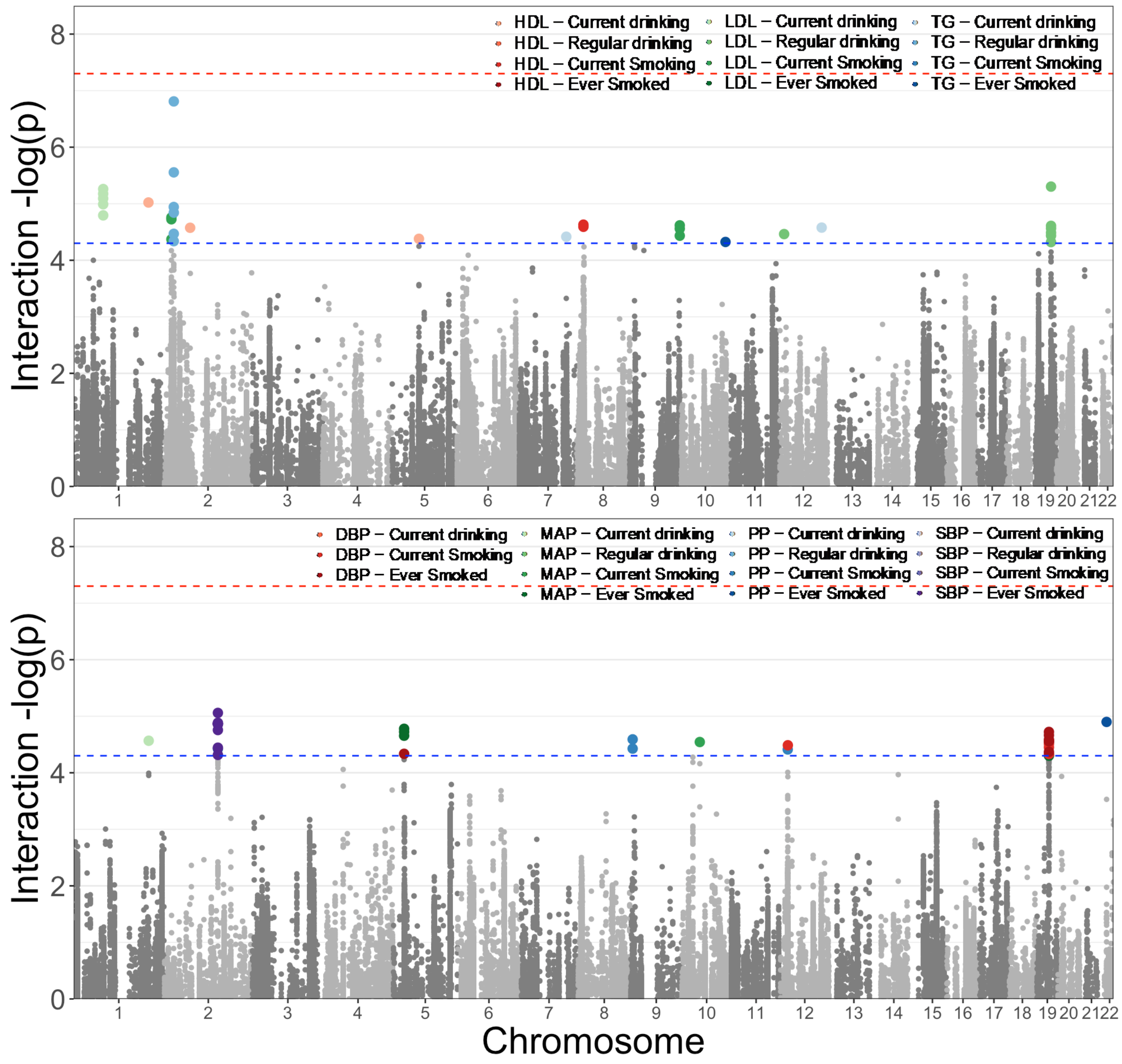

HDL – Current drinking
HDL – Regular drinking
HDL – Current Smoking
HDL – Ever Smoked
LDL – Current drinking
LDL – Regular drinking
LDL – Current Smoking
LDL – Ever Smoked
TG – Current drinking
TG – Regular drinking
TG – Current Smoking
TG – Ever Smoked
DBP – Current drinking
DBP – Current Smoking
DBP – Ever Smoked
MAP – Current drinking
MAP – Regular drinking
MAP – Current Smoking
MAP – Ever Smoked
PP – Current drinking
PP – Regular drinking
PP – Current Smoking
PP – Ever Smoked
SBP – Current drinking
SBP – Regular drinking
SBP – Current Smoking
SBP – Ever Smoked
Interaction -log(p)
0
2
4
6
8
1 2 3 4 5 6 7 8 9 10 11 12 13 14 15 16 17 18 19 20 21 22
Chromosome

E.

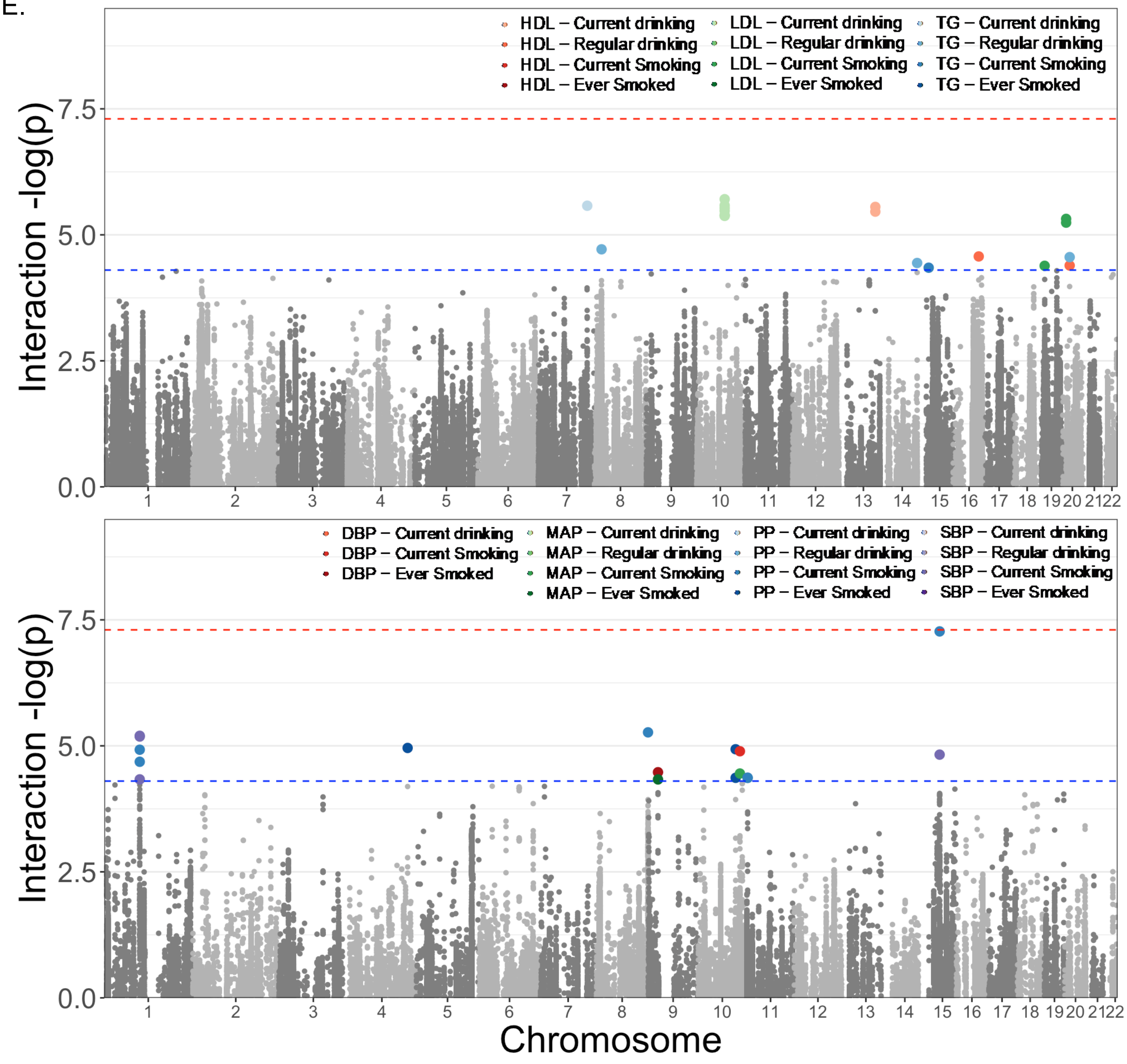
HDL – Current drinking
HDL – Regular drinking
HDL – Current Smoking
HDL – Ever Smoked
LDL – Current drinking
LDL – Regular drinking
LDL – Current Smoking
LDL – Ever Smoked
TG – Current drinking
TG – Regular drinking
TG – Current Smoking
TG – Ever Smoked
Interaction -log(p)
7.5
5.0
2.5
0.0
1 2 3 4 5 6 7 8 9 10 11 12 13 14 15 16 17 18 19 20 21 22
DBP – Current drinking
DBP – Current Smoking
DBP – Ever Smoked
MAP – Current drinking
MAP – Regular drinking
MAP – Current Smoking
MAP – Ever Smoked
PP – Current drinking
PP – Regular drinking
PP – Current Smoking
PP – Ever Smoked
SBP – Current drinking
SBP – Regular drinking
SBP – Current Smoking
SBP – Ever Smoked
Interaction -log(p)
7.5
5.0
2.5
0.0
1 2 3 4 5 6 7 8 9 10 11 12 13 14 15 16 17 18 19 20 21 22
Chromosome

Supplemental Figure 2. (A) Regional Plot *GCNT4* × Current Smoking and HDL (African Ancestry); (B) Summary of Relevant Data

A.

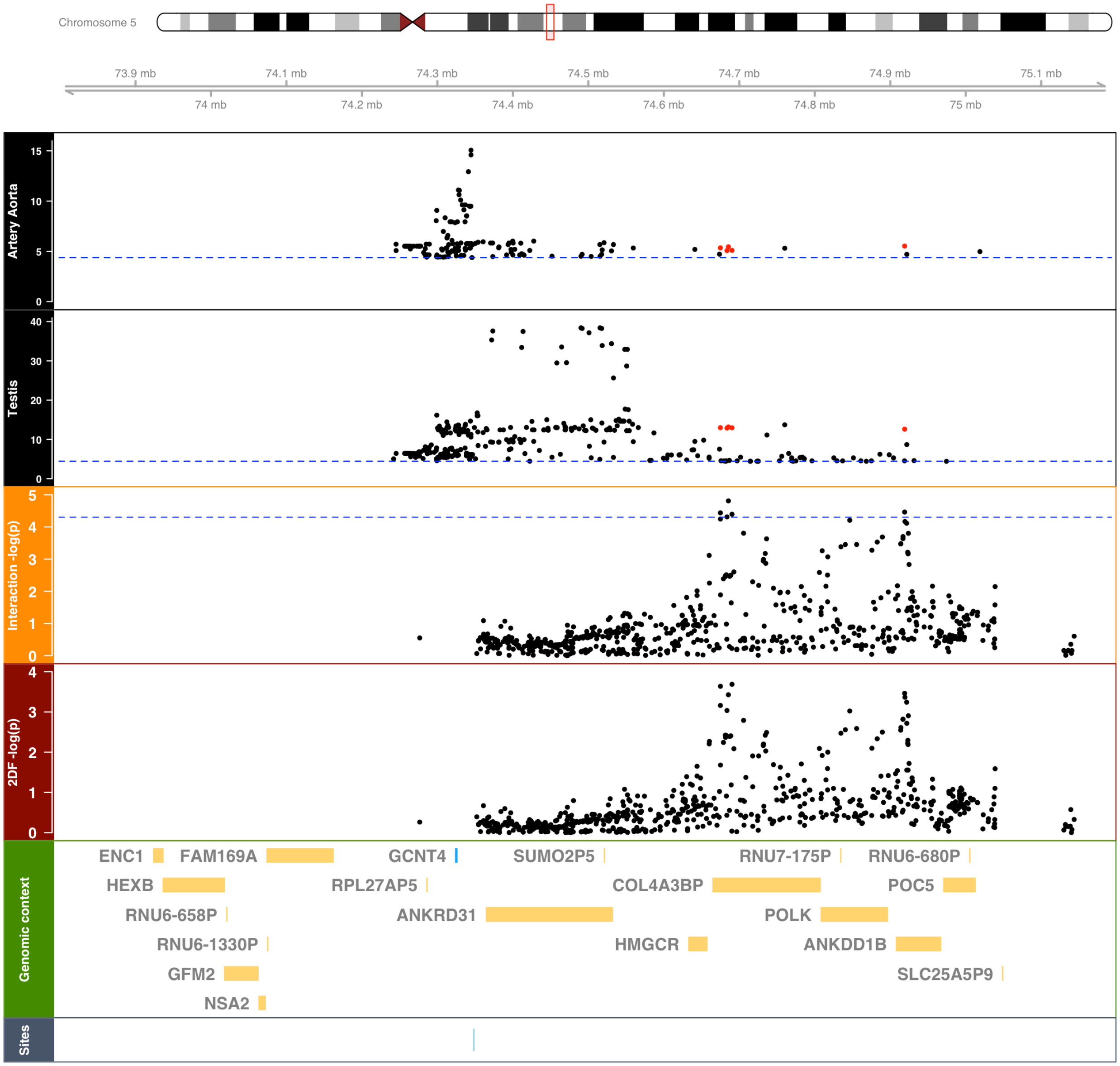

B.

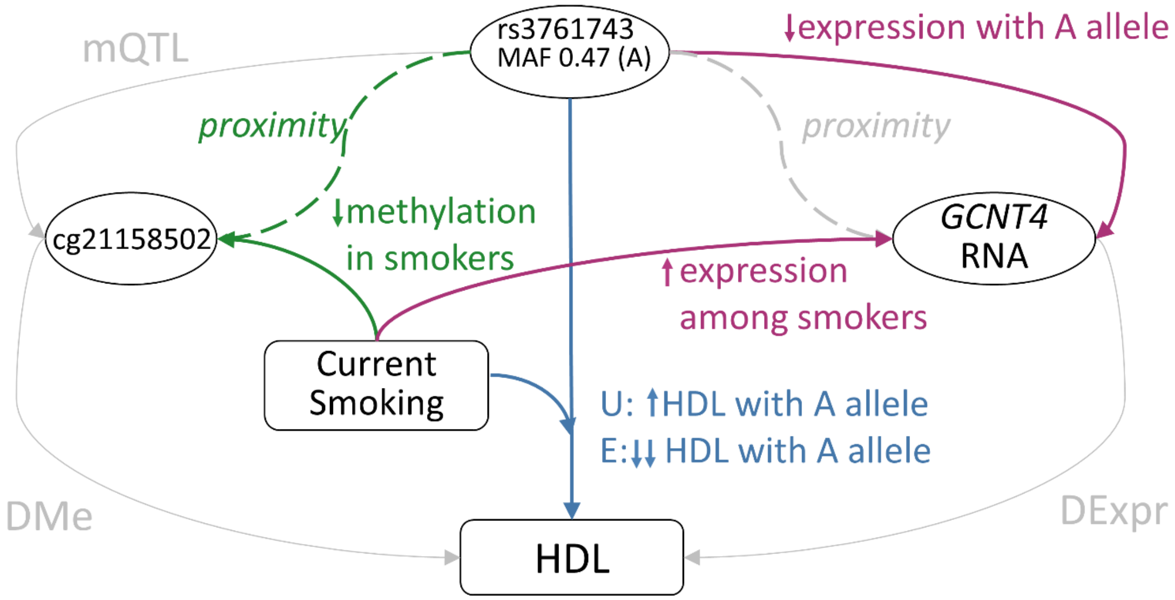
rs3761743
MAF 0.47 (A)
mQTL
proximity
proximity
↓expression with A allele
cg21158502
↓methylation
in smokers
GCNT4
RNA
↑expression
among smokers
Current
Smoking
U: ↑HDL with A allele
E:↓↓ HDL with A allele
DMe
DExpr
HDL

Supplemental Figure 3. (A) Regional Plot *PTPRZ1* × Ever Smoked and HDL (African Ancestry); (B) Summary of Relevant Data

A.

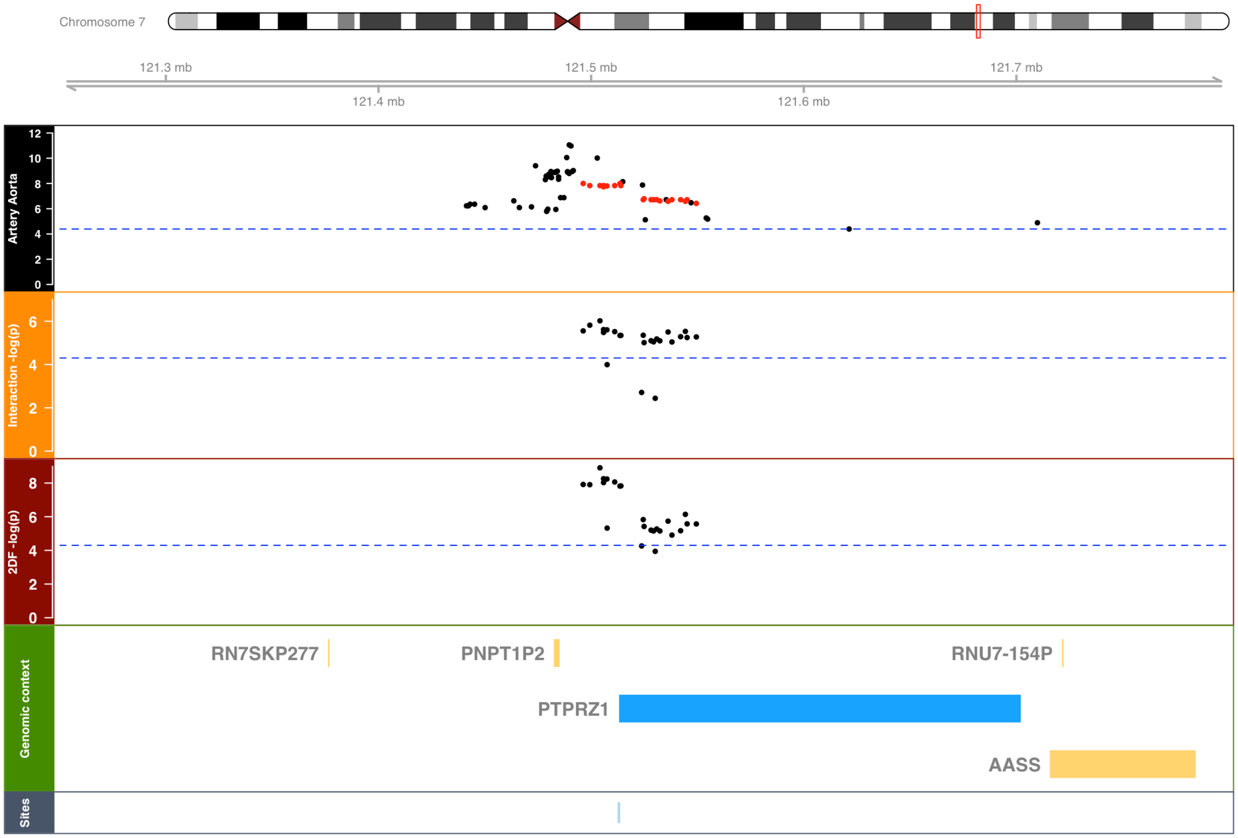

B.

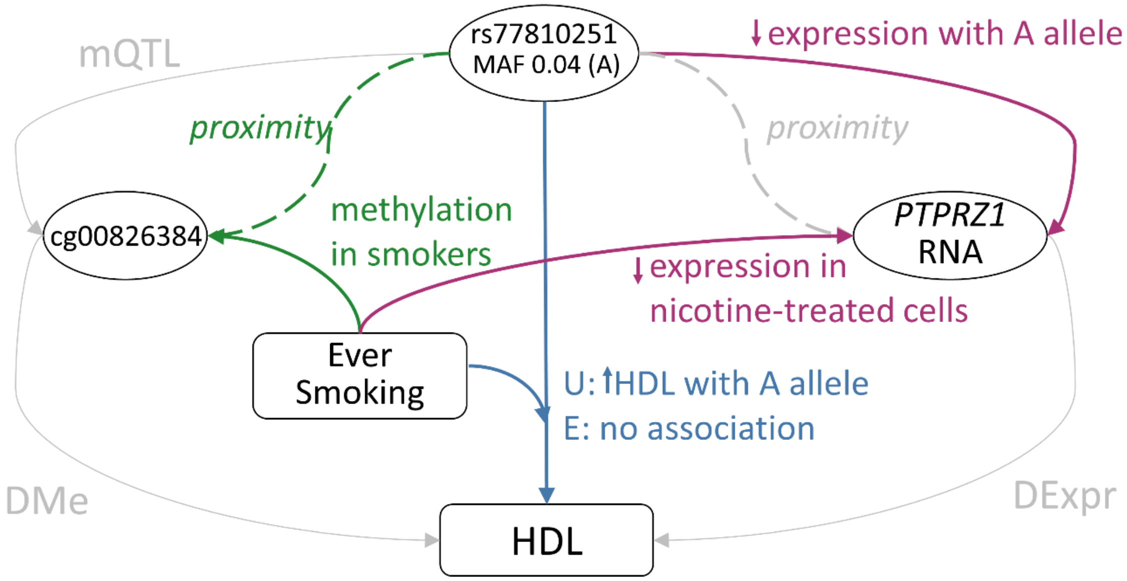
rs77810251
MAF 0.04 (A)
mQTL
proximity
↓expression with A allele
proximity
cg00826384
methylation
in smokers
PTPRZ1
RNA
↓expression in
nicotine-treated cells
Ever
Smoking
U: ↑HDL with A allele
E: no association
DMe
DExpr
HDL

Supplemental Figure 4. (A) Regional Plot *SYN2* × Current Smoking on PP (African Ancestry); (B) Summary of Relevant Data

A.

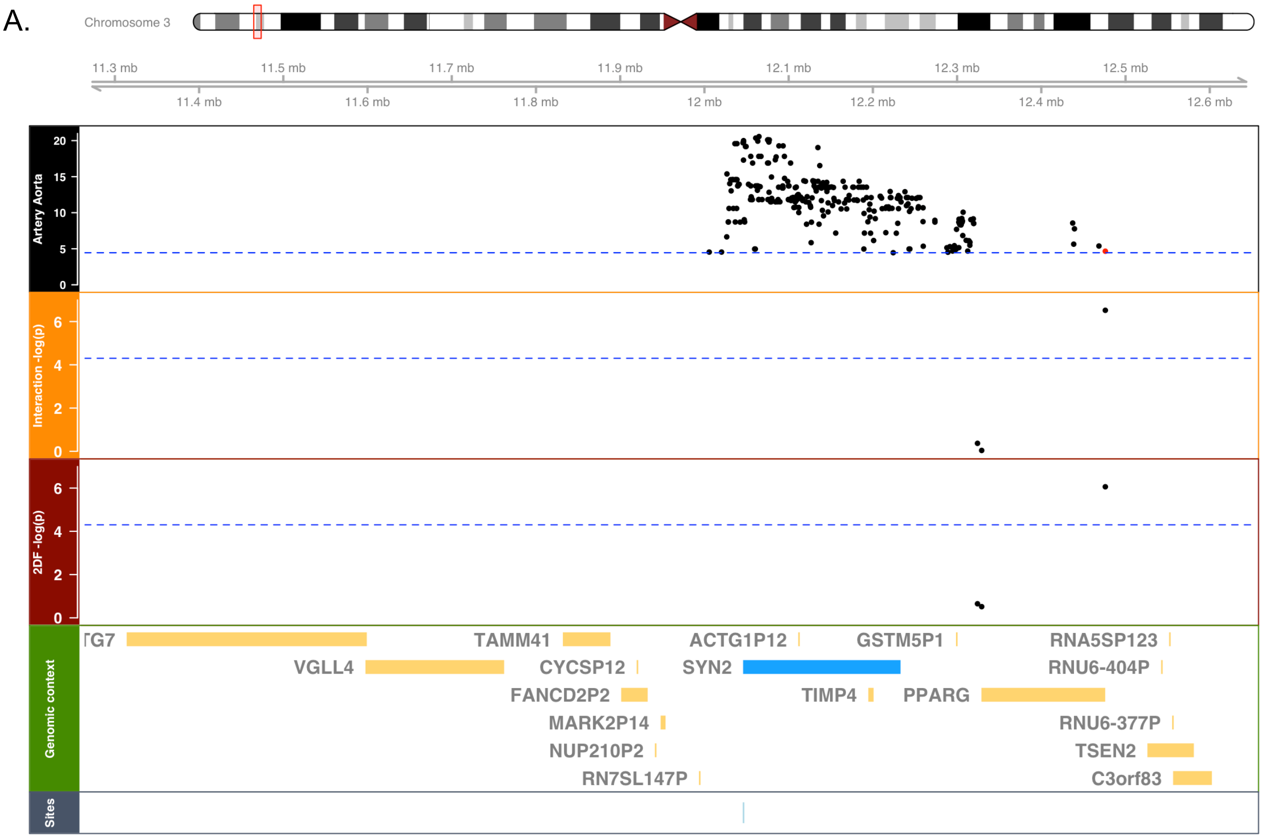

B.

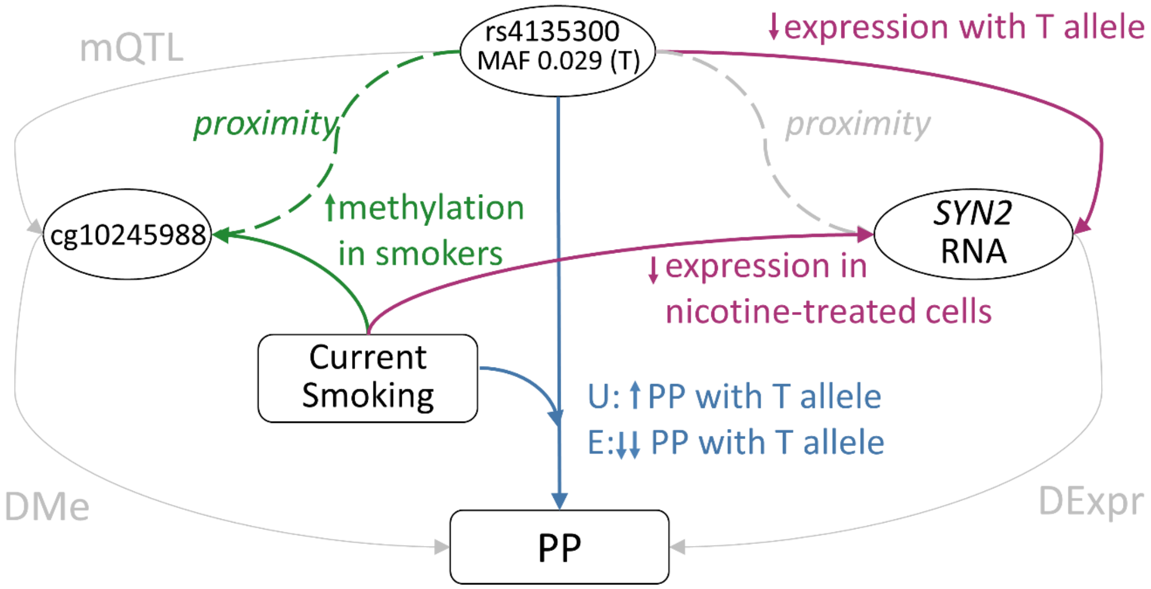

rs4135300
MAF 0.029 (T)
mQTL
proximity
proximity
↓expression with T allele
cg10245988
↑methylation
in smokers
SYN2
RNA
↓expression in
nicotine-treated cells
Current
Smoking
U: ↑PP with T allele
E:↓↓ PP with T allele
DMe
DExpr
PP

Supplemental Figure 5. (A) Regional Plot *ALDH2* × Ever Smoked and MAP (Asian Ancestry); (B) Summary of Relevant Data

A.

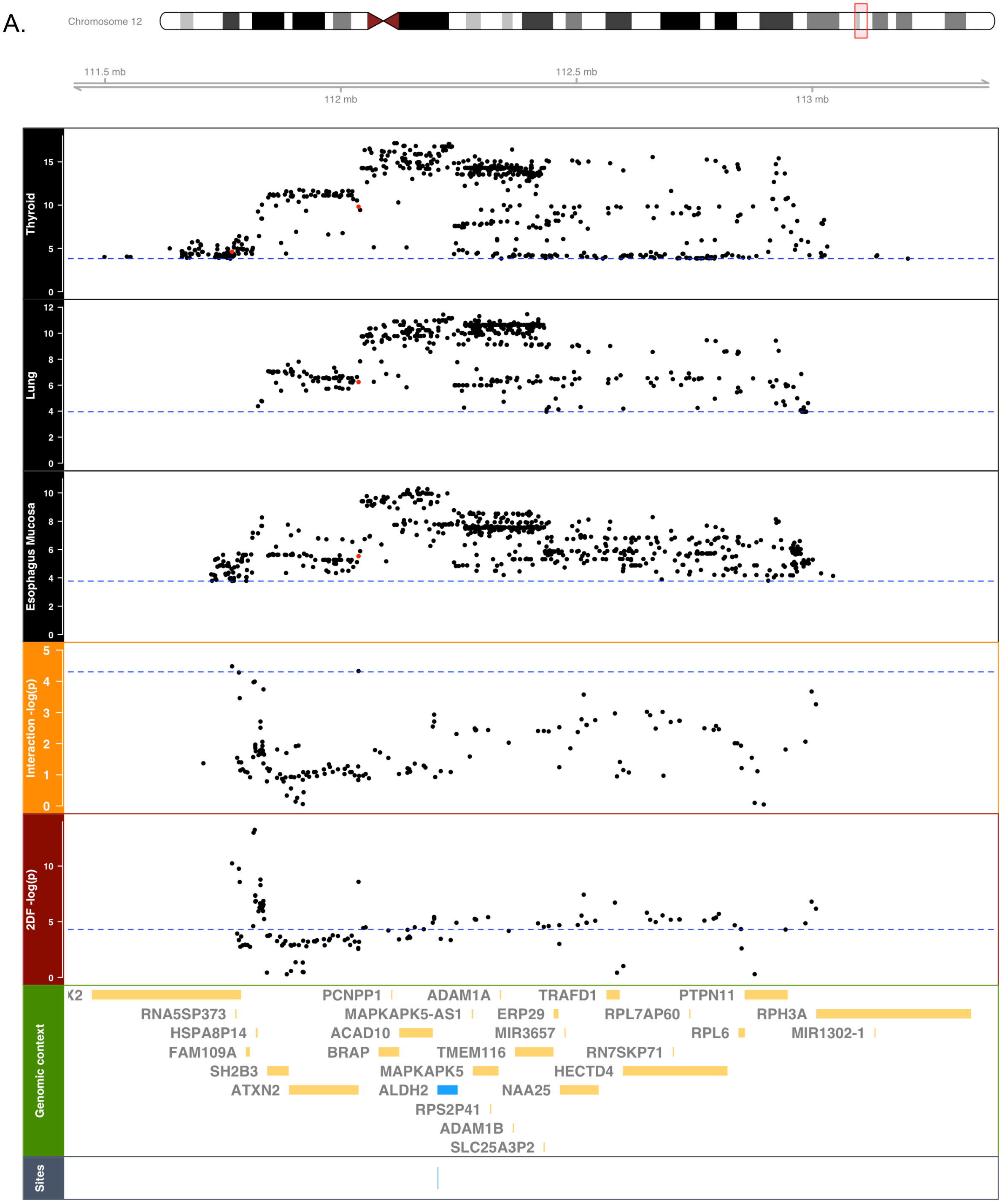

B.

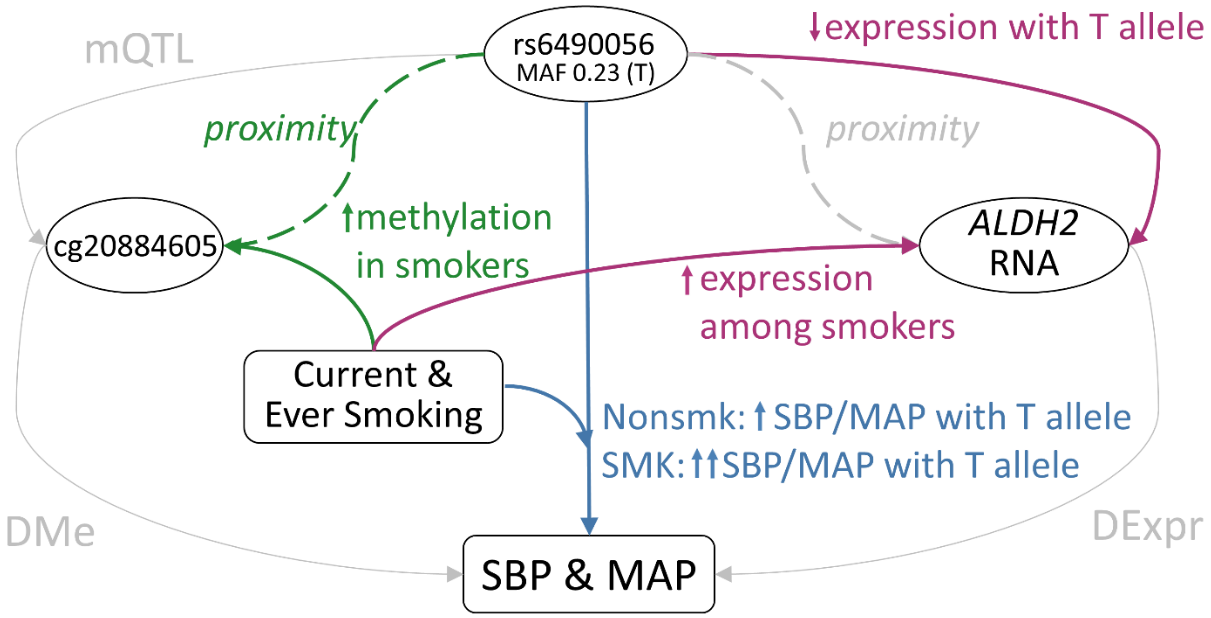

rs6490056
MAF 0.23 (T)
↓expression with T allele
mQTL
proximity
proximity
cg20884605
↑methylation
in smokers
ALDH2
RNA
↑expression
among smokers
Current &
Ever Smoking
Nonsmk: ↑SBP/MAP with T allele
SMK: ↑↑SBP/MAP with T allele
DMe
DExpr
SBP & MAP

Supplemental Figure 6. Regional Plot *ALDH2* × Current Smoking and SBP (Asian Ancestry)

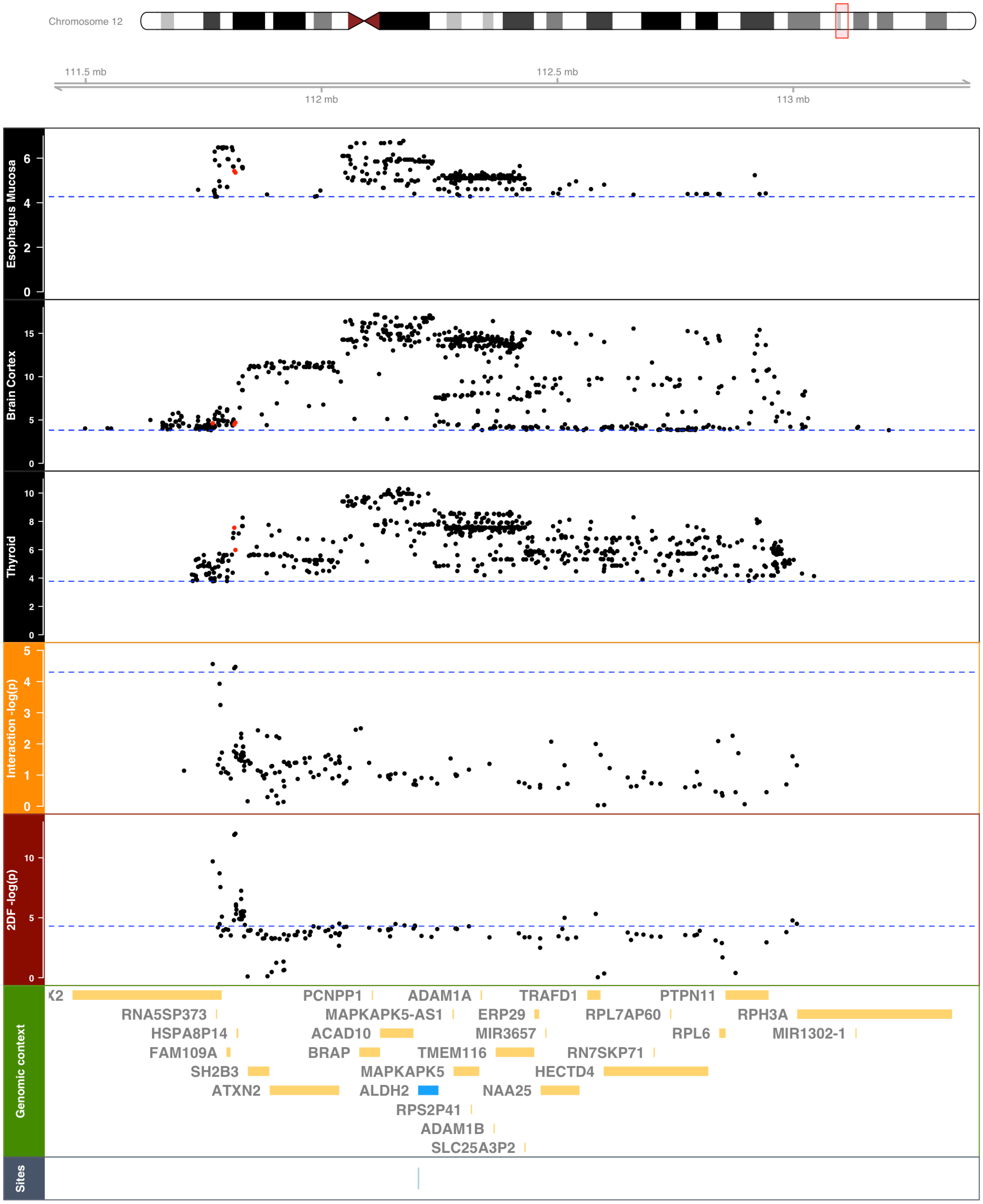

Supplemental Figure 7. Regional Plot *ALDH2* × Ever Smoked and SBP (Asian Ancestry)

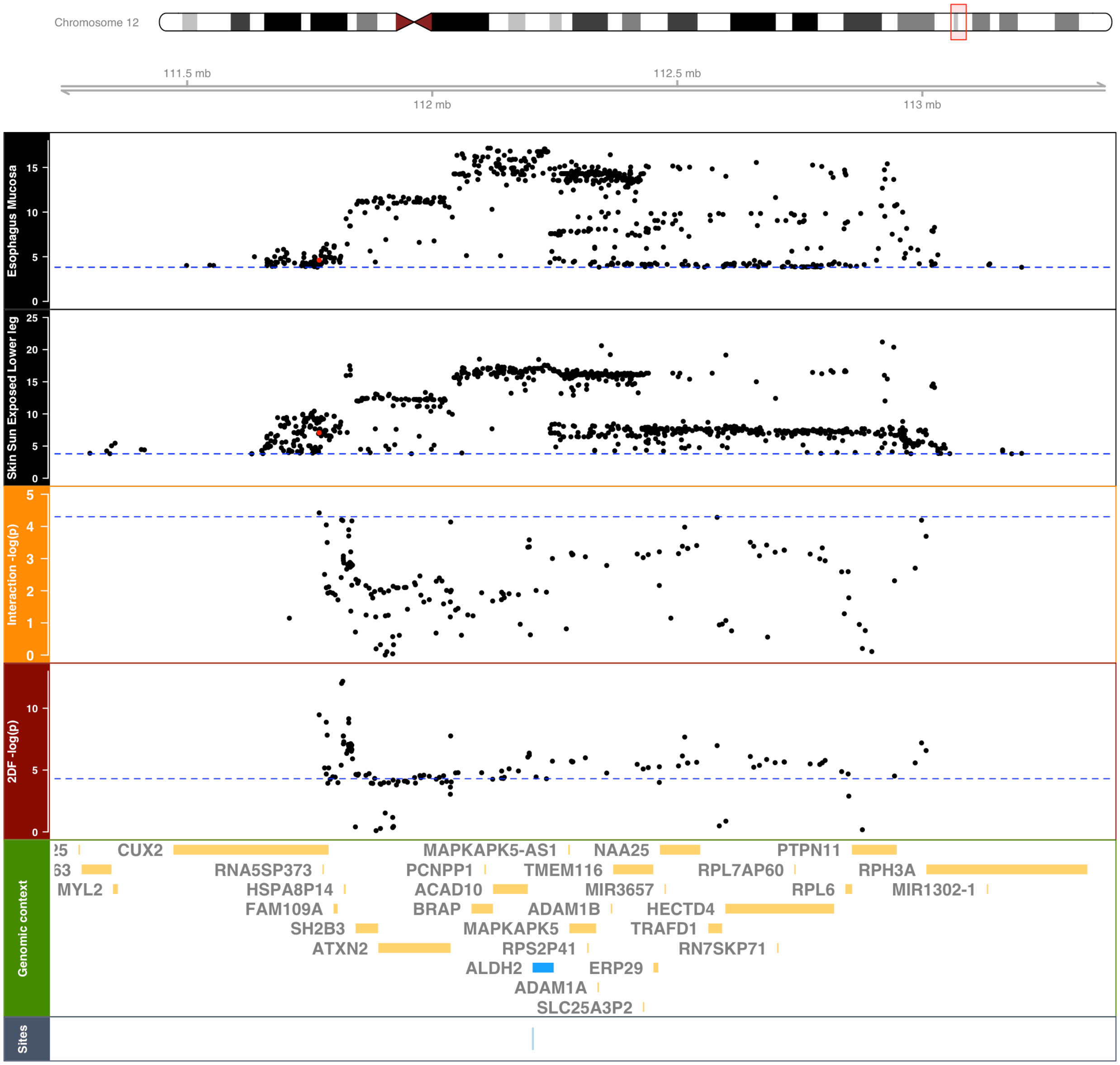

Supplemental Figure 8. (A) Regional Plot *TMEM116* × Ever Smoked and MAP (Asian Ancestry); (B) Summary of Relevant Data

A.

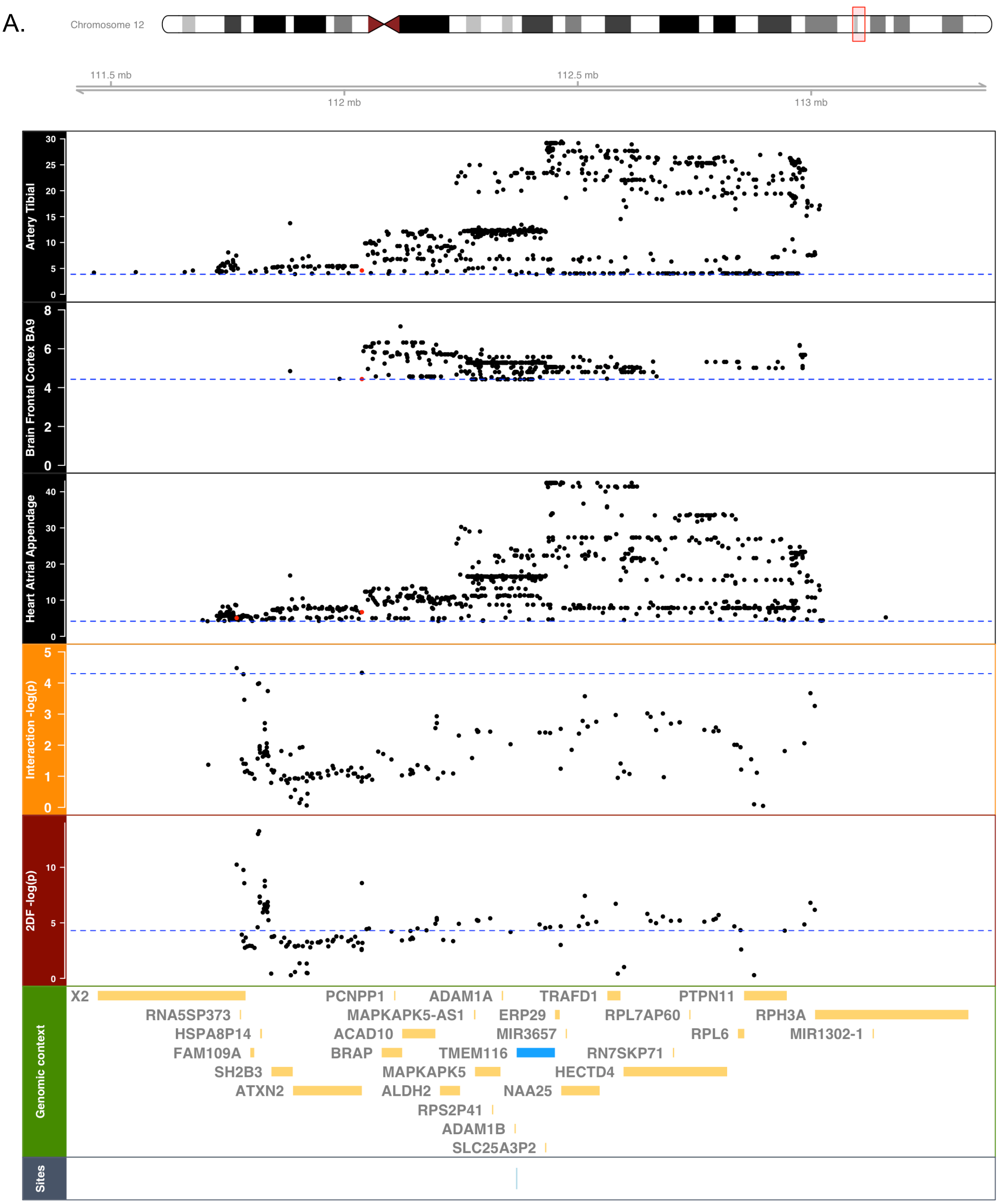

B.

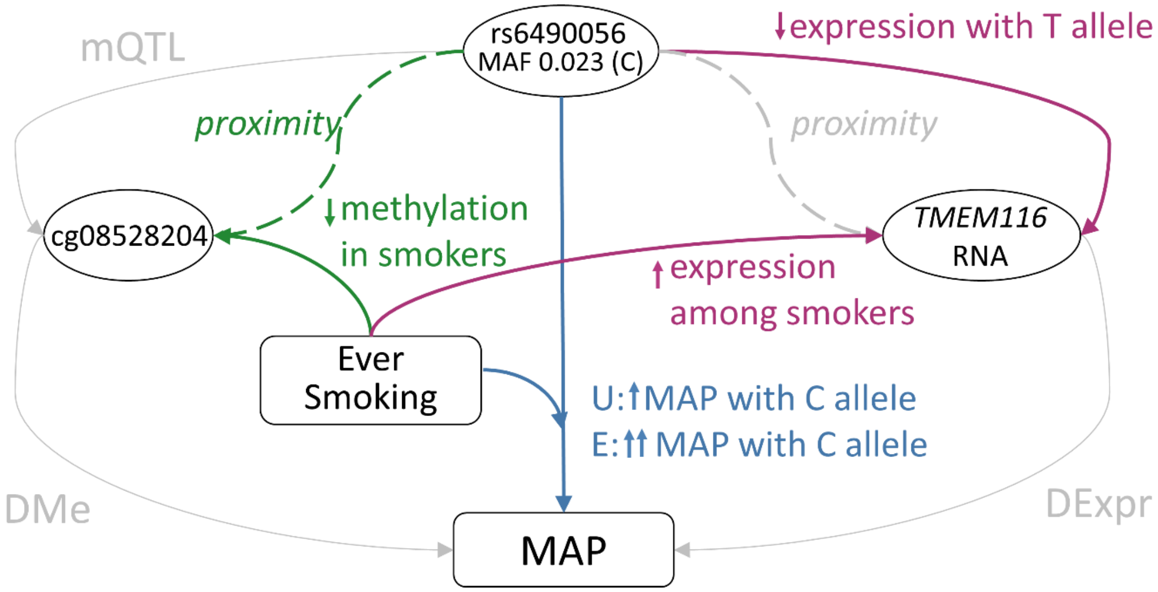

mQTL
rs6490056
MAF 0.023 (C)
↓expression with T allele
proximity
proximity
cg08528204
↓methylation
in smokers
TMEM116
RNA
↑expression
among smokers
Ever
Smoking
U:↑MAP with C allele
E:↑↑MAP with C allele
DMe
DExpr
MAP
